# A Three-Axis Conceptual Framework for Dietary Risk: Universal, Age–Generational and Sex-Specific Deviations from Dietary Recommendations in a Cross-Sectional Study of Adults

**DOI:** 10.3390/nu18213644

**Published:** 2026-11-01

**Authors:** Dmitry B. Nikityuk, Olga G. Bogdanova, Irina A. Lapik, Inna Yu. Tarmaeva

**Affiliations:** 1 Federal Research Centre of Nutrition, Biotechnology and Food Safety, 2/14 Ustinsky Proyezd, Moscow 109240, Russia; nikitjuk@ion.ru (D.B.N.); tarmaeva@ion.ru (I.Y.T.); 2 I.M. Sechenov First Moscow State Medical University (Sechenov University), Ministry of Health of the Russian Federation, Moscow 119991, Russia; 3 East-Siberian Institute of Medical and Ecological Research, 3, Microdistrict 12a, Angarsk 665826, Russia; olga.bogdanova2001@gmail.com

**Keywords:** dietary risk, three-axis framework, generational differences, sex differences, nutrient adequacy, added sugars, sugar-sweetened beverages, dietary fibre, sodium, dietary patterns, cross-sectional study

## Abstract

**Background/Objectives**: Dietary risk factors account for a substantial share of the non-communicable disease burden, and generation theory offers a lens for explaining why eating patterns differ between people socialised under different economic and food-supply conditions. Data on generational and sex differences in the habitual diet remain scarce. We hypothesised that dietary risk in this population is structured along three axes—universal, age–generational and sex-specific—each potentially requiring a different preventive approach. This study aimed to characterise differences in energy, macronutrient, micronutrient and food-group intake between generations and between sexes, to quantify the adequacy of intake against national (MR 2.3.1.0253-21) and WHO reference values, to identify which deviations are universal, which differ between birth-year groups and which are sex-specific, and to propose corresponding levels of preventive action. **Methods**: We performed a cross-sectional analysis of habitual dietary intake in 1601 adults (405 men, 1196 women; age 18–76 years) recruited during preventive examinations and community screenings in the Republic of Buryatia and the Irkutsk Region in three survey waves in 2022–2024. Habitual intake was quantified with a validated semi-quantitative food-frequency questionnaire covering 60 food items and yielding daily intakes of energy, macronutrients, fatty-acid classes, added sugars, dietary fibre, minerals, vitamins and 14 food groups. Participants were classified by birth year into three generations: Baby Boomers (1943–1963, *n* = 140), Generation X (1964–1984, *n* = 915) and Generation Y/Z (1985–2005, *n* = 546). Intakes were compared with the Russian methodological recommendations MR 2.3.1.0253-21 and with WHO recommendations. Group comparisons used the Kruskal–Wallis test, with Dunn post-hoc tests and Bonferroni correction, and the Mann–Whitney U test; Benjamini–Hochberg FDR correction was applied across outcomes. Generational trends were additionally modelled on rank-transformed outcomes with adjustments for survey site. Dietary patterns were derived via principal component analysis of energy-adjusted food-group intakes. Reporting follows the STROBE-nut guidelines. With 1601 participants, the study had >80% power to detect |ρ| ≥ 0.07. **Results**: Three patterns emerged. Universal: total fat exceeded 30%E in 99.8% of participants (median 41.5–42.5%E in men, 46.5–48.0%E in women), saturated fatty acids exceeded 10%E in 95.1%, reported dietary fibre was below 20 g/day in 99.3% (median 6.1–6.6 g/day), although the composition data were shown to reflect crude rather than total dietary fibre, salt equivalent exceeded 5 g/day in 93.4%, the Na:K molar ratio exceeded 1 in 98.9% and calcium was below the requirement in 75.6%. Age–generational: discretionary sugars differed markedly between birth-year groups—the proportion of sugar-sweetened beverage (SSB) consumers rose from 52.2% to 74.7% across generations in men (+22.6 percentage points, +43% relative) and from 20.2% to 51.7% in women (+31.5 points, +156% relative; both *p* < 0.001, q < 0.001; rank-biserial r = 0.38 and 0.33), mean SSB intake reached 78.4 g/day in Generation Y/Z men and the proportion of men exceeding the 10%E limit for added sugars rose more than threefold, from 8.7% to 32.1% (*p* = 0.001); in men, the intake of bread, pasta and cereals (397 → 362 g/day) and starch fell across generations, while vitamin C rose (77.2 → 106.8 mg/day, *p* = 0.004). Principal component analysis identified a “sweetened-beverage and discretionary foods” pattern whose score increased monotonically from older to younger generations in both sexes (men Δ = +0.64 z-units, *p* < 0.001; women Δ = +0.41, *p* = 0.002); the gradient persisted after an adjustment for survey site, within each survey wave and after restriction to plausible energy reporters. Sex-specific: reported iron intake fell below the sex-specific reference value in 74.9–77.7% of women versus 4.1–7.9% of men, despite the higher absolute intake in men; men reported higher absolute energy, protein, bread product, potato, sodium and iron intake, while women reported higher fat and polyunsaturated fatty acid contribution to energy and higher vegetable intake; differences in fruit, confectionery and vitamin C did not persist after an adjustment for survey site. **Conclusions**: In this adult population, the classical dietary risks—excess saturated fat, excess salt and low calcium—were shared by all birth-year groups and both sexes, whereas the intake of sugar-sweetened beverages and added sugars was concentrated in adults born after 1985; because the design is cross-sectional, this difference may reflect age, birth cohort or both. These findings are consistent with a conceptual three-axis framework of dietary risk—universal, age–generational and sex-specific—in which each axis may call for a different preventive approach: population-wide measures addressing fat, salt, calcium and fish; age-targeted measures addressing sweetened beverages in younger adults; and, in women, an evaluation of the low reported iron intake against biochemical markers of iron status in future studies. The framework was derived from a regional sample and requires external validation; it could be tested in other populations.

## 1. Introduction

Improving population health and increasing life expectancy remain priority objectives of state policy in many countries [1,2,3,4]. Nutrition makes a substantial contribution to the formation of health: up to 50% of the factors that determine human health and working capacity are estimated to be related to the character of the diet [5,6]. Disturbances in the structure of the diet are associated with 30–50% of cases of chronic non-communicable disease, including obesity, cardiovascular disease, type 2 diabetes mellitus and several types of cancer [6,7]. The adult working-age population is exposed to a combination of dietary and environmental risk factors, which contributes to the rising prevalence of nutrition-related conditions nationally [8,9,10] and internationally [3,11].

One productive approach to the analysis of eating behaviour is generation theory, according to which each generation is formed under unique socio-economic and climatic-geographical conditions and acquires relatively stable models of consumption, including in the sphere of nutrition [12,13]. Members of older generations more often display adherence to traditional foods and home cooking, whereas younger generations, socialised in the era of digitalisation and of an expanding market of ultra-processed products, are more inclined towards fast food, sweetened beverages and energy-dense foods [14,15]. Understanding these differences is important for the development of differentiated preventive strategies that take the generational profile of the target group into account [16]. At the same time, in cross-sectional data, differences between generations cannot be separated from differences in age, so generation theory can serve as an interpretive frame for such data but not as a causal model.

Contemporary research in nutrition science actively examines the relationship among nutrient intake, dietary patterns and the risk of nutrition-related disease, including cardiometabolic and multimorbidity outcomes [17,18]. Particular attention is paid to the assessment of actual macro- and micronutrient intake, its correspondence with physiological requirements and the identification of sex differences in the structure of the diet [19,20]. Nevertheless, data on generational and sex features of the diet remain limited. National and cross-national dietary surveys have documented inadequate intakes of fibre, calcium, iron and vitamin D in adult populations [21,22,23], but a comprehensive analysis combining the generational approach with a quantitative assessment of nutrient adequacy remains scarce.

The novelty of the present study lies in three aspects: the combination of the generational approach with a quantitative assessment of nutrient adequacy in a population for which such data are scarce; the use of current national reference values of MR 2.3.1.0253-21 [24], with age- and sex-specific differentiation, alongside the corresponding WHO criteria [25]; and the separation of the observed deviations into three distinct axes of dietary risk—universal, age–generational and sex-specific—proposed here as a conceptual framework, each axis of which may imply a different preventive instrument and a different level of dietary assessment. We hypothesised that dietary risk in this population is structured along these three axes rather than being uniformly distributed.

Accordingly, the aim of the present study was to evaluate differences in the intake of energy, macro- and micronutrients and of the principal food groups between generations and sexes in an adult population sample and to compare the values obtained with national (MR 2.3.1.0253-21) and WHO reference values [24]. The objectives were to characterise the habitual intake of energy, macronutrients, micronutrients and 14 food groups by generation and sex; to quantify the adequacy of that intake against national and WHO reference values; to identify which deviations are uniform across the population and which follow a generational or a sex-specific structure; and to propose, on that basis, hypotheses regarding the corresponding levels of preventive action and of dietary assessment.

## 2. Materials and Methods

### 2.1. Study Design and Participants

This was a cross-sectional study of habitual dietary intake in the adult population of the Baikal region of Russia. Data were collected in nine municipal districts of the Republic of Buryatia (Bichursky, Ivolginsky, Kabansky, Kyakhtinsky, Mukhorshibirsky, Pribaikalsky, Selenginsky and Tarbagataysky districts and the city of Ulan-Ude) and in one adjacent district of the Irkutsk Region between 2022 and 2024, in three survey waves: the city of Ulan-Ude in 2022 (*n* = 620), the district of the Irkutsk Region in 2023 (*n* = 69) and the eight rural districts of Buryatia in 2024 (*n* = 912). Participants were recruited during preventive medical examinations and community health screenings; participation was voluntary. Recruitment was not based on probability sampling, and persons attending preventive examinations may differ from the general population in health awareness, health status and socio-economic position. Because each survey wave corresponds to a single site or group of sites, the survey period and place of residence cannot be separated, and an adjustment for survey site (Section 2.6) also adjusts for survey wave; these issues are considered in Section 4.5.

Inclusion criteria were age 18 years or older at the time of the survey, permanent residence in the study area and a completed dietary questionnaire with a recorded date of birth. Exclusion criteria were age below 18 years, pregnancy or lactation and an incomplete questionnaire that did not allow energy intake to be computed.

### 2.2. Generational Classification

Participants were assigned to generations by year of birth in accordance with the periodisation used in Russian applications of generation theory [12]: Baby Boomers, born 1943–1963 (*n* = 140); Generation X, born 1964–1984 (*n* = 915); and Generation Y/Z, born 1985–2005 (*n* = 546). Because the design is cross-sectional, generation and chronological age are collinear, and the gradients reported below cannot be decomposed into birth-cohort and ageing components. We therefore use the term “generational” throughout in a descriptive sense—as a difference between groups defined by year of birth—and not as a claim about a cohort effect. Where the direction of a difference is consistent with a known age pattern, as it is for overweight and obesity, this is noted explicitly. Although data were collected over 2022–2024, each participant was surveyed once and the collection window was short; an age–period–cohort decomposition requires repeated cross-sections spanning substantially longer periods and, even then, additional identifying assumptions [26], and it was therefore not attempted. Accordingly, the second axis of the proposed framework is referred to as “age–generational”, and the term “generational” is used as shorthand for differences between birth-year groups.

### 2.3. Dietary Assessment

Dietary intake over the previous month was assessed by a trained interviewer using a semi-quantitative food-frequency questionnaire (FFQ) containing 60 food items with standard portion sizes. The questionnaire is based on the food-frequency method developed at the Federal Research Centre of Nutrition, Biotechnology and Food Safety for the assessment of habitual diets in the Russian population; its development and the evaluation of its validity are described in [27]. Like other food-frequency instruments, it is designed primarily to rank individuals by intake rather than to estimate absolute intake precisely [28], and the absolute values reported below are interpreted with this in mind. Reported frequencies and portions were converted to mean daily intake in grams and then to nutrient intakes using Russian food-composition tables. Dietary fibre values were taken as given in these tables; because the tables were compiled over several decades with different analytical methods, the values for some foods may not correspond to total dietary fibre as determined using current enzymatic–gravimetric methods; this was examined directly (Section 2.6, Section 3.10 and Section 4.1). The output comprised energy; protein, total fat and total carbohydrate; saturated, poly-unsaturated, *n*-6 and *n*-3 fatty acids; cholesterol; mono- and disaccharides, added sugars and starch; dietary fibre; sodium, potassium, calcium, magnesium, phosphorus and iron; vitamins A, B1, B2, niacin and C; and the mass consumed per day of 14 food groups (bread, pasta and cereals; potatoes; vegetables; fruit; meat and meat products; fish and fish products; milk and dairy products; eggs; confectionery; vegetable oil; butter; table sugar; sugar-sweetened beverages; tea and coffee). Reporting of the dietary assessment follows the STROBE-nut recommendations for nutritional epidemiology [29]. Questionnaires with missing items were not imputed: an item left blank for a food that the respondent reported never consuming was treated as zero intake, and records from which total energy intake could not be computed were excluded at the inclusion stage. In the analytic sample, there were no missing values for any nutrient or food-group variable, and only one missing value for height and body mass index. Salt equivalent was derived from sodium as Na (g) × 2.54. The Na:K molar ratio was computed as (Na mg/22.99)/(K mg/39.10), where 22.99 and 39.10 are the atomic masses of sodium and potassium, respectively.

### 2.4. Anthropometry

Height and body weight were measured using standard procedures, and body mass index (BMI) was calculated as weight (kg)/height (m)^2^ and classified according to MR 2.3.1.0253-21 and the WHO classification: underweight < 18.5, normal 18.5–24.9, overweight 25.0–29.9 and obesity ≥ 30.0 kg/m^2^ [24]. The basal metabolic rate was estimated using the Mifflin–St Jeor equations as specified in MR 2.3.1.0253-21 and used to compute the ratio of reported energy intake to the basal metabolic rate (EI:BMR) as a plausibility check. Values below 1.35 and above 2.4 were taken to indicate probable under- and over-reporting, respectively, following the approach of Goldberg et al. [30,31]. Because self-reported intake is subject to systematic misreporting [32], EI:BMR was used to characterise reporting quality and to define a sensitivity analysis, not to correct the intake values.

### 2.5. Reference Values and Definition of Adequacy

Intakes were compared with two sets of criteria, which are distinguished throughout: the Russian methodological recommendations on the norms of physiological requirements for energy and nutrients, MR 2.3.1.0253-21 [24], and the food-based recommendations of the WHO.

The following were taken from MR 2.3.1.0253-21 [24]. Age- and sex-specific reference values for energy, protein, total fat and total carbohydrate for the age bands 18–29, 30–44, 45–64 and 65–74 years, assigned to the second physical-activity group (physical-activity coefficient 1.6). This group was chosen because it corresponds to the predominantly non-manual and light-manual occupations of the districts surveyed and is the category applied by default to adult populations in MR 2.3.1.0253-21; neither occupational nor leisure-time physical activity was recorded, so the same coefficient was applied to all participants. This assumption determines the absolute reference values for energy, protein, fat and carbohydrate, but not the indicators expressed as a percentage of energy or per 1000 kcal, nor the micronutrient reference values; its consequences are considered in Section 4.5. Reference values for adults are as follows: dietary fibre 20–25 g/day; calcium 1000 mg/day (1200 mg/day from 65 years); phosphorus 700 mg/day; magnesium 420 mg/day; potassium 3500 mg/day; sodium 1300 mg/day as the physiological requirement with an upper limit of 2000 mg/day; iron 10 mg/day for men and 18 mg/day for women; vitamin A 900 µg RE/day for men and 800 µg RE/day for women; thiamine 1.5 mg/day; riboflavin 1.8 mg/day; niacin 20 mg NE/day; and vitamin C 100 mg/day. Limits for the contribution of macronutrients to energy are as follows: total fat ≤ 30%E, saturated fatty acids ≤ 10%E, polyunsaturated fatty acids 6–10%E, *n*-6 5–8%E, *n*-3 1–2%E with an optimal *n*-6:*n*-3 ratio of 5–10:1, protein 12–14%E, carbohydrate 56–58%E, added sugars < 10%E (<5%E in persons with BMI ≥ 25 kg/m^2^) and dietary cholesterol ≤ 300 mg/day.

Two food-based criteria were taken from the WHO recommendations: vegetables plus fruit ≥ 400 g/day and salt < 5 g/day. Fish intake was additionally assessed against the equivalent of two servings per week (<40 g/day). Following the approach used previously by our group [16], intake below 80% of the reference value was classified as below the reference value (potentially inadequate intake) and above 120% as above the reference value (potentially excessive intake). These categories describe reported intake relative to reference values and do not establish nutritional deficiency. The reference values of MR 2.3.1.0253-21 are set to cover the requirements of almost all healthy individuals in a group, so intake below them indicates an increased probability of inadequacy rather than inadequacy itself [33]; the nutritional status, including the iron status, can be established only via biochemical and clinical assessment [34], which was not performed in this study.

### 2.6. Statistical Analysis

Analyses were performed in Python 3.12 with pandas, SciPy 1.13, statsmodels and scikit-learn. Normality was assessed via the Shapiro–Wilk test; all dietary variables departed from normality and are reported as the median and interquartile range, Me [Q1; Q3]. Differences between the three generations within each sex were tested via the Kruskal–Wallis test followed by Dunn post-hoc tests with Bonferroni correction; differences between sexes within each generation were tested via the Mann–Whitney U test. Categorical variables were compared via the chi-square test.

Benjamini–Hochberg false discovery rate correction was applied across the set of dietary outcomes tested within each sex; the resulting q values are given for the generational (Kruskal–Wallis) comparisons and are quoted in the text alongside the corresponding *p* values. The post-hoc Dunn tests were Bonferroni-corrected and were not additionally FDR-adjusted. The q values for the sex comparisons (Mann–Whitney U) are not reported, since those tests were not part of the FDR family. The categorical criteria form a separate family and were not FDR-corrected; they are reported with uncorrected *p* values. Because a large number of outcomes were tested in each sex, statistical significance alone is not treated as evidence of nutritional relevance: for the key findings, the absolute and relative differences in intake and the rank-biserial correlation, as a measure of effect size, are reported alongside *p* and q.

Because the distribution of survey sites differed across generations, generational trends were additionally estimated in linear models fitted to rank-transformed outcomes with generation entered as an ordinal term and survey site as a covariate, and a sensitivity analysis was repeated in the rural-district subsample alone. The same rank-based models, with survey site and generation as covariates, were used to test whether the sex differences persisted after an adjustment for place of residence. A second sensitivity analysis was restricted to participants whose ratio of reported energy intake to estimated basal metabolic rate fell between 1.35 and 2.4, that is, excluding probable under- and over-reporters.

Dietary patterns were derived via principal component analysis of the energy-adjusted food-group intakes (expressed per 1000 kcal and standardised); components were retained on the basis of the scree plot and interpretability, and component scores were compared between generations and sexes. Spearman rank correlation was used for the correlation analysis, and correlations with |ρ| < 0.20 were treated as weak and are not interpreted as associations. Confidence intervals for the mean dietary-pattern score were obtained via bootstrap resampling with 3000 replicates. Two-sided *p* < 0.05 was taken as statistically significant.

With 1601 participants, the study had more than 80% power, at a two-sided α of 0.05, to detect a Spearman correlation of |ρ| ≥ 0.07 in the total sample and a standardised difference of d ≥ 0.16 between men and women. In the smallest generational comparison—Baby Boomer men (*n* = 46) versus Generation Y/Z men (*n* = 190)—80% power corresponded to d ≥ 0.46 or, for a binary criterion, to a rise in the proportion affected from 8.7% to about 26%; the corresponding comparison in women (*n* = 94 versus *n* = 356) retained 80% power for d ≥ 0.32. The study was therefore well powered for the population-wide comparisons but not for small generational differences in the least numerous subgroups, and the absence of a significant difference in those subgroups is not evidence of equivalence. Because all generational comparisons were performed separately within each sex, the predominance of women in the sample reduces the precision of the estimates for men but does not bias the within-sex comparisons.

Additional analyses addressed specific sources of bias. Because the survey wave coincided with the survey site (Section 2.1), the generational gradient in sugar-sweetened beverages was re-estimated within each survey wave separately and in rank-based models with the survey wave in place of the survey site; the proportion of consumers was also modelled based on logistic regression adjusted for the survey site, and 95% confidence intervals for the differences in proportions between Generation Y/Z and Baby Boomers were calculated using the Newcombe method. Differential misreporting was examined by comparing the EI:BMR distribution between generations within each sex, and the universal deviations were re-examined among plausible reporters. To establish whether the dietary fibre values of the composition data correspond to total dietary fibre, the fibre content implied for each food group was estimated via linear regression without the intercept of dietary fibre intake on the intakes of bread, pasta and cereals, potatoes, vegetables, fruit, meat, dairy products and fish; the coefficients, expressed per 100 g, were compared with the total dietary fibre content of the corresponding foods [35]. The contribution of fat to energy was recalculated after the exclusion of fat from vegetable oil. The sensitivity of energy adequacy to the physical-activity assumption was examined by comparing reported energy intake with individually estimated requirements (BMR × PAL) at PAL values of 1.4, 1.6 and 1.9. For iron, the proportion of women aged 51 years and over with intake below 8 mg/day, the recommended intake for postmenopausal women in the Dietary Reference Intakes [33], was calculated alongside the national reference value.

### 2.7. Ethics

The study was conducted in accordance with the Declaration of Helsinki. Ethical approval was obtained from the Local Ethics Committee of the Federal State Budgetary Scientific Institution “East-Siberian Institute of Medical and Ecological Research” (FSBSI ESIMER) (Protocol No. 1, approved 27 January 2022). All participants provided written informed consent prior to the survey.

## 3. Results

### 3.1. Characteristics of the Study Population

Table 1 presents the characteristics of the study population. The analytic sample comprised 1601 adults, of whom 405 (25.3%) were men and 1196 (74.7%) women; the median age was 45 [36; 53] years. The median BMI was 26.3 [23.4; 29.7] kg/m^2^, and 61.0% of participants were overweight or obese (60.0 % of men and 61.4% of women). The prevalence of overweight and obesity fell markedly from older to younger generations in both sexes (men 78.2% in Baby Boomers versus 47.6% in Generation Y/Z, *p* < 0.001; women 72.4% versus 41.0%, *p* < 0.001), a difference that, in a cross-sectional design, reflects the age structure of the generations.

The ratio of reported energy intake to estimated basal metabolic rate was 1.52 [1.27; 1.90] overall (men 1.47, women 1.56); 32.5% of participants reported an intake below 1.35 × BMR, indicating probable under-reporting, and 7.1% above 2.4 × BMR, indicating probable over-reporting; in total, 39.6% of participants had EI:BMR values outside the plausible range. Absolute intakes reported below should therefore be interpreted with caution, whereas indicators expressed as a percentage of energy or per 1000 kcal are less sensitive to the misreporting of total intake.

### 3.2. Energy and Macronutrients

Energy and macronutrient intakes are shown in Table 2. Median energy intake was higher in men than in women in every generation (*p* < 0.001) and differed modestly between generations (men *p* = 0.037, women *p* = 0.033), being highest in Generation X in both sexes. The most striking feature of the macronutrient profile was the contribution of fat to energy: 41.5–42.5%E in men and 46.5–48.0%E in women, with women exceeding men in every generation (all *p* < 0.001). Saturated fatty acids provided 13.4–14.1%E and did not differ between generations in either sex. The contribution of carbohydrate was correspondingly low (37.7–42.0%E), and protein contributed 11.8–12.4%E. Median dietary fibre intake was 6.1–6.6 g/day, or 2.5–3.1 g per 1000 kcal, against a reference of 20–25 g/day or 10 g per 1000 kcal; as shown in Section 3.10, these values were derived from composition data that correspond to crude rather than total dietary fibre. Added sugars were higher in the younger generations of both sexes, although after FDR correction, this reached significance only for the absolute intake of women (24.6 → 35.9 g/day, *p* = 0.005, q = 0.035); the corresponding contribution to energy did not survive correction in either sex (men 5.5 → 7.4%E, *p* = 0.032, q = 0.078; women 4.5 → 6.6%E, *p* = 0.011, q = 0.066). Starch intake fell across generations in both sexes and did survive correction (men *p* < 0.001, q = 0.002; women *p* = 0.001, q = 0.013).

### 3.3. Micronutrients

Micronutrient intakes are shown in Table 3. Sodium intake corresponded to a salt equivalent of 9.4–10.5 g/day in men and 7.9–8.4 g/day in women, approximately twice the WHO limit of 5 g/day, and was lower in the youngest generation of men than in Generation X (9.4 versus 10.5 g/day, *p* < 0.001, q = 0.005). Potassium intake was below the reference value of 3500 mg/day in most participants, and the Na:K molar ratio was close to 2 in all groups. Median calcium intake ranged from 700 to 810 mg/day against a reference of 1000–1200 mg/day, and the calcium-to-phosphorus ratio was 0.58–0.70 against an optimal ratio of 1:1, falling below 1:1 in 99.7% of participants. Iron intake was higher in men (16.9–18.9 mg/day) than in women (13.6–14.1 mg/day) in every generation (*p* < 0.001), but the sex-specific reference values of 10 and 18 mg/day mean that the resulting adequacy picture is reversed. Thiamine, riboflavin and niacin intakes were below the reference values in the majority of participants in all groups. Several minerals and B vitamins differed nominally across generations in men—potassium, calcium, magnesium, phosphorus, the Ca:P ratio, thiamine and riboflavin, all with p between 0.013 and 0.035—but none of these comparisons survived FDR correction (q = 0.057–0.079) and they are not interpreted further. Vitamin C intake increased across generations in men (77.2 → 106.8 mg/day, *p* = 0.004, q = 0.023) but did not differ between generations in women, in whom it was substantially higher than in men throughout (*p* < 0.001).

### 3.4. Food Groups

**Figure 1:**
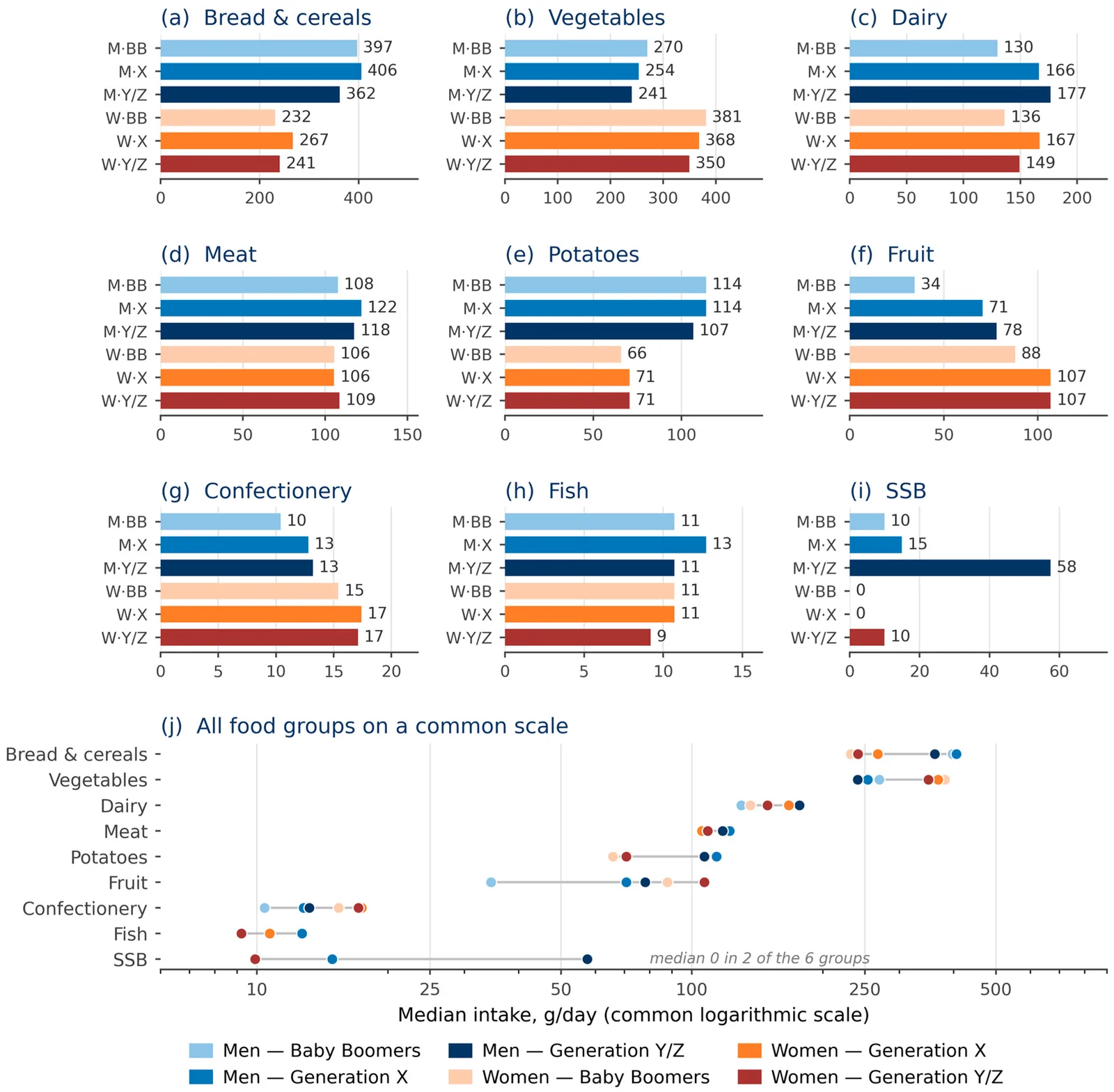
Median food-group intake by generation and sex. Panels (**a**–**i**) are small multiples with a separate horizontal scale for each food group; each panel contains the six sex-by-generation groups in a fixed order, and the data labels give the median intake in g/day. Separate scales are used because the medians span two orders of magnitude, from 9 g/day for fish to 406 g/day for bread and cereals, so values in panels (**a**–**i**) should be compared within, not across, panels. Panel (**j**) places all nine food groups on a single common logarithmic scale so that their magnitudes can be compared directly: each row shows the six sex-by-generation medians, joined by a line spanning the lowest and the highest of them, and the food groups are ordered by their overall median. M, men; W, women; BB, Baby Boomers; X, Generation X; Y/Z, Generation Y/Z; SSB, sugar-sweetened beverages. Interquartile ranges and *p* and q values are given in Table 4.

Food-group intakes are shown in Table 4 and Figure 1. Men consumed substantially more bread, pasta and cereals than women (362–406 versus 232–267 g/day, all *p* < 0.001) and more potatoes, whereas women consumed more vegetables (350–381 versus 241–270 g/day), more fruit and more confectionery (all *p* < 0.001). The consumption of bread, pasta and cereals declined in the youngest generation of men (397 → 362 g/day, *p* < 0.001, q = 0.005); vegetable intake showed the same direction but did not survive FDR correction (270 → 241 g/day, *p* = 0.014, q = 0.057). Fish intake was uniformly low, with medians of 9–13 g/day in all groups, and was lowest in the youngest generation of both sexes (men p = 0.009, q = 0.050; women *p* = 0.003, q = 0.023). The intake of sugar-sweetened beverages showed the steepest generational gradient of any food group in both sexes (*p* < 0.001), and table sugar intake rose across generations in women (*p* = 0.002, q = 0.016). The deviations from reference values implied by these food-group intakes are quantified in Section 3.5.

### 3.5. Adequacy of Intake Relative to National and Who Reference Values

**Figure 2:**
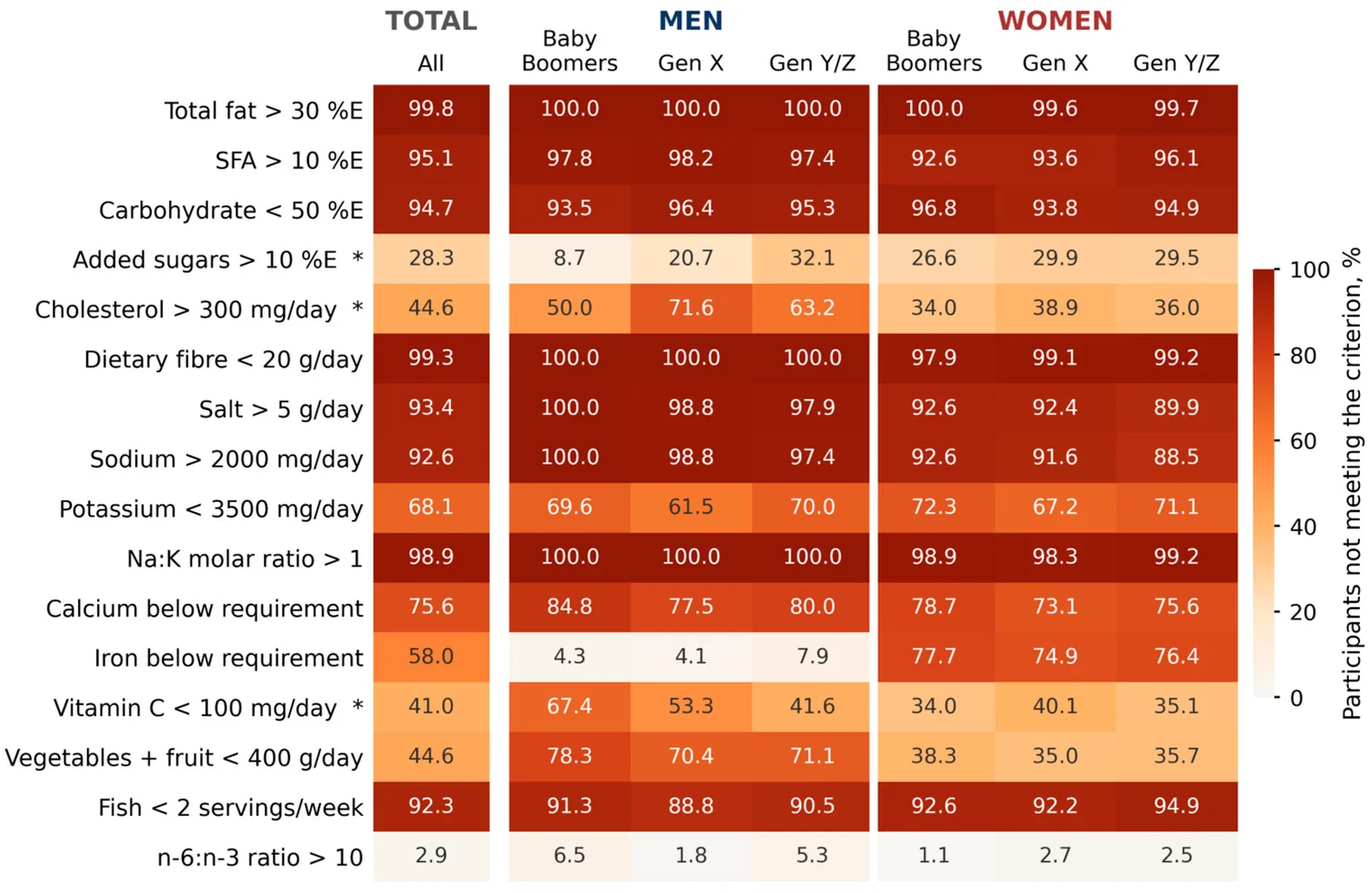
Proportion of participants (%) not meeting national (MR 2.3.1.0253-21) and WHO dietary criteria, by generation and sex, shown as a heatmap of the values given in Table 6. Cell values are percentages of the group concerned, and the colour scale runs from 0% (pale) to 100% (dark); the first column refers to the whole sample. Criteria marked with an asterisk (*) differed significantly across generations within one sex (added sugars, cholesterol and vitamin C, all in men; chi-square *p* ≤ 0.018). The fat, saturated-fat, carbohydrate, dietary-fibre, salt, sodium, Na:K and fish criteria are failed by almost everyone in every group; added sugars is the only criterion with a pronounced generational gradient, and iron the only one that separates the sexes. SFA, saturated fatty acids; %E, percentage of total daily energy.

**Figure 3:**
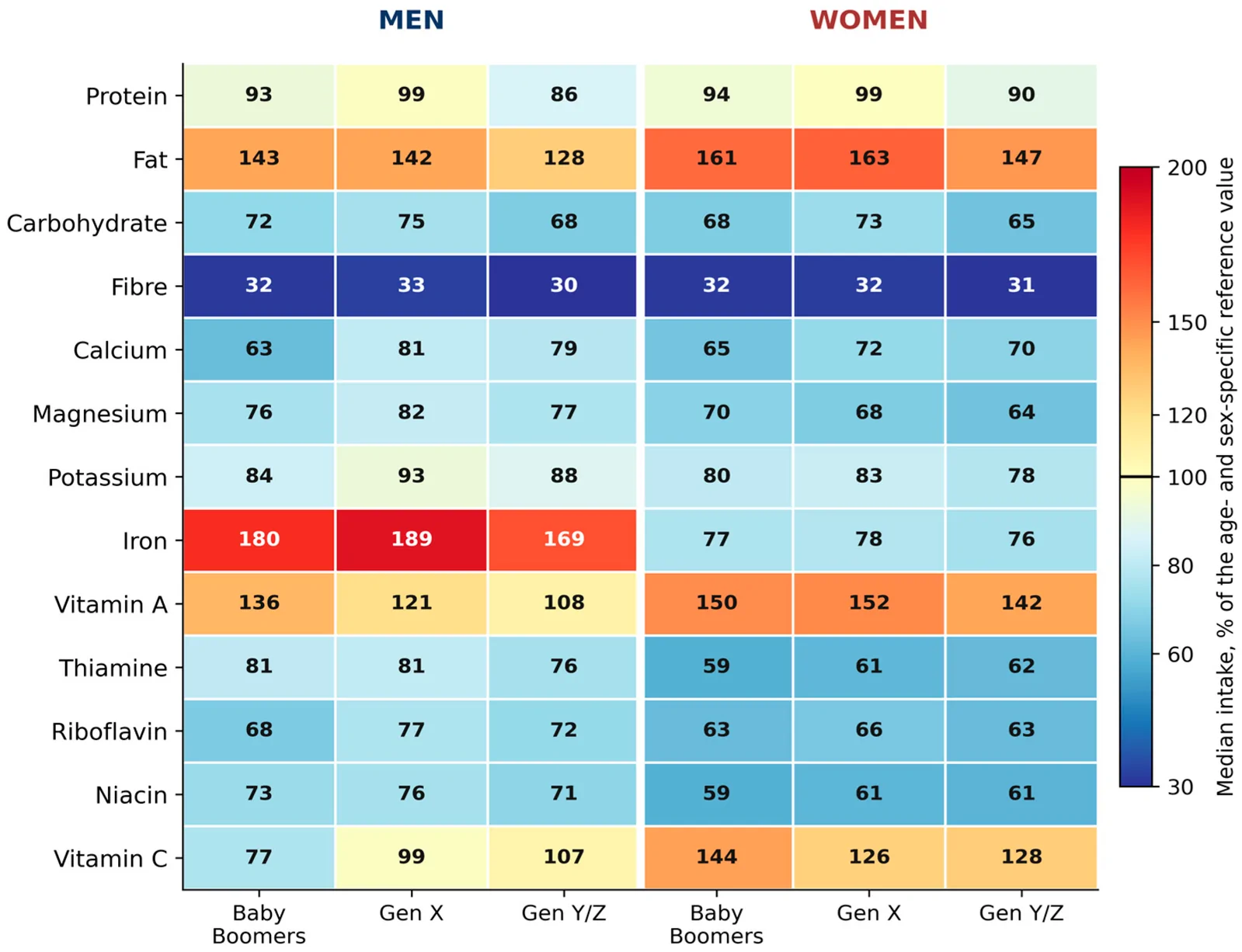
Median habitual intake expressed as a percentage of the age- and sex-specific reference value of MR 2.3.1.0253-21, by generation and sex. Cell values are medians, in per cent of the reference value; the colour scale is centred on 100%, so blue denotes a shortfall and red an excess, and the black mark on the colour bar indicates full adequacy. In both sexes, the profile is characterised by a pronounced excess of fat and a deep shortfall of dietary fibre (30–33% of the reference value), with calcium, magnesium, riboflavin and niacin between 60% and 90%. The reversal in iron is visible as the contrast between 169–189% in men and 76–78% in women.

The proportions of participants with intake below 80% or above 120% of the age- and sex-specific reference value are given in Table 5, and the proportions failing specific national and WHO criteria are given in Table 6 and Figure 2. Dietary fibre was below 80% of the reference value in 94.7–100% of participants in every generation and sex, the most extreme shortfall observed, although this proportion is inflated by the crude-fibre basis of the composition data (Section 3.10). Total fat exceeded 120% of the reference value in 56.8–84.2% and phosphorus in 68.1–97.8%. Calcium was below 80% of the reference in 47.3–76.1%, magnesium in 45.6–72.3%, riboflavin in 53.3–68.5% and niacin in 60.9–84.0%. Thiamine intake below 80% of the reference value was markedly more common in women (74.8–83.0%) than in men (43.8–57.9%). The clearest sex asymmetry concerned iron: reported intake fell below 80% of the reference value in 52.1–53.9% of women and in only 1.2–2.2% of men, whereas 86.3–94.1% of men exceeded 120% of their reference value. The overall adequacy profile of each generation and sex, expressed as the median intake relative to the reference value, is summarised in Figure 3.

Against the criteria in Table 6, deviation was near-universal and largely independent of the generation: total fat exceeded 30%E in 99.8% of participants, saturated fatty acids exceeded 10%E in 95.1%, carbohydrate was below 50%E in 94.7%, dietary fibre was below 20 g/day in 99.3%, salt equivalent exceeded 5 g/day in 93.4%, sodium exceeded 2000 mg/day in 92.6% and the Na:K molar ratio exceeded 1 in 98.9%. Polyunsaturated fatty acids exceeded 10%E in 78.5%, and magnesium fell below the reference in 86.6%, thiamine in 90.9%, riboflavin in 83.7% and niacin in 93.8%. Fish intake was below the equivalent of two servings per week in 92.3%. Vegetable and fruit intake fell below 400 g/day in 70.4–78.3% of men but in only 35.0–38.3% of women. Two criteria did differ by generation, both in men: the proportion exceeding the 10%E limit for added sugars rose from 8.7% in Baby Boomers to 20.7% in Generation X and 32.1% in Generation Y/Z (*p* = 0.001), while the proportion with vitamin C intake below 100 mg/day fell from 67.4% to 41.6% (*p* = 0.003). Among participants with BMI ≥ 25 kg/m^2^, for whom MR 2.3.1.0253-21 recommends added sugars below 5%E, 59.8% exceeded that limit.

### 3.6. Generational Gradient in Added Sugars and Sugar-Sweetened Beverages

**Figure 4:**
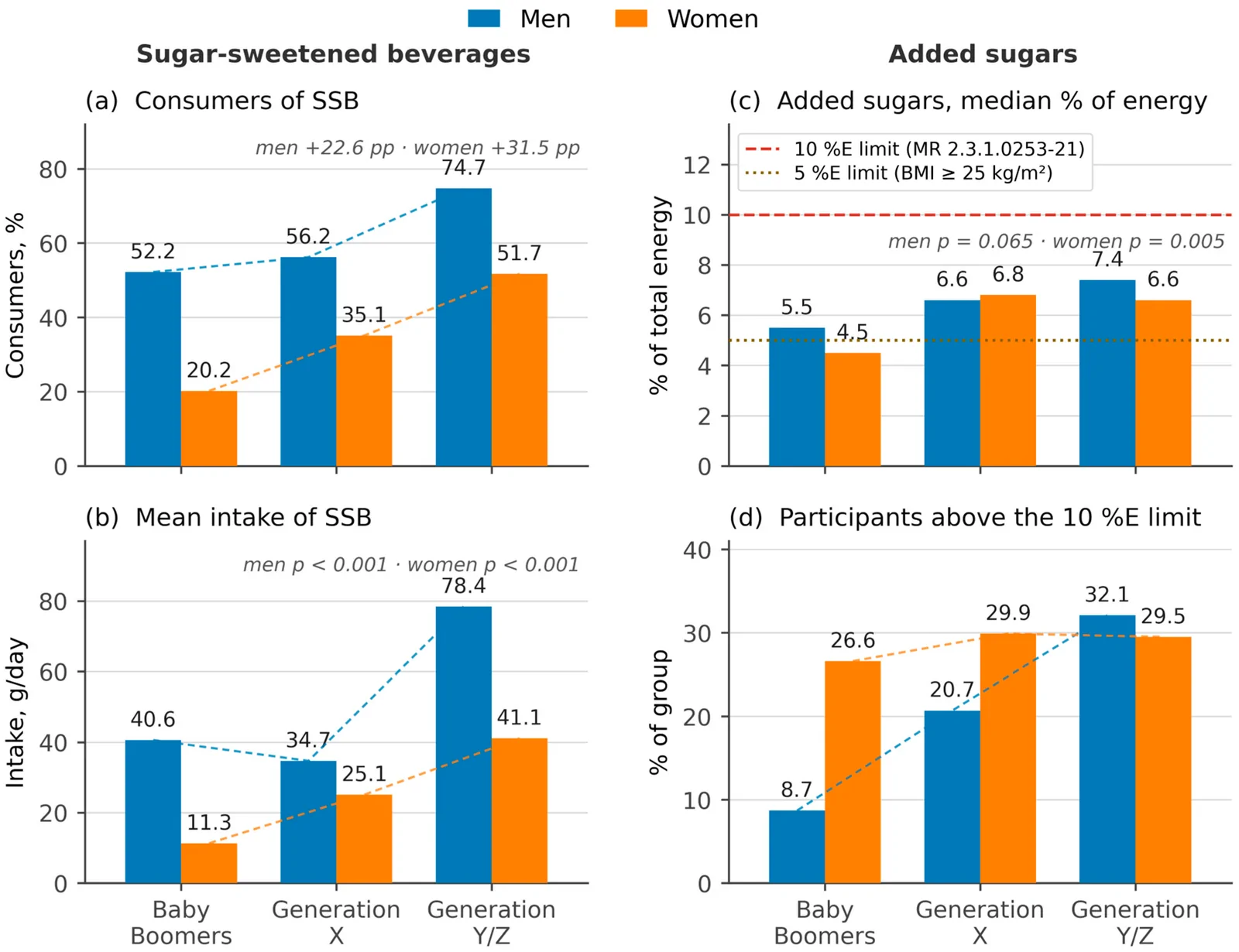
Sugar-sweetened beverages and added sugars across generations, by sex. The four panels are arranged in two blocks, sugar-sweetened beverages on the left and added sugars on the right; panels within a block share a common generation axis and each panel has a single vertical scale. (**a**) Proportion of consumers of sugar-sweetened beverages. (**b**) Mean intake of sugar-sweetened beverages in the whole group; the mean in Baby Boomer men exceeds that in Generation X men because the few consumers in the oldest group reported unusually large amounts, whereas the proportion of consumers and the median intake rise monotonically. (**c**) Median intake of added sugars as a percentage of total energy, with the 10%E limit of MR 2.3.1.0253-21 (dashed line) and the 5%E limit recommended for persons with a body mass index of 25 kg/m^2^ or above (dotted line). (**d**) Proportion of each group exceeding the 10%E limit. Dashed connectors join the bars of the same sex and indicate the direction of the generational gradient; *p* values are from the Kruskal–Wallis test across generations within sex. SSB, sugar-sweetened beverages; pp, percentage points; %E, percentage of total daily energy.

Figure 4 presents the generational gradient in sugar-sweetened beverages and added sugars. The intake of sugar-sweetened beverages was the single dietary variable with the strongest and most consistent generational gradient. In men, the proportion of consumers rose from 52.2% in Baby Boomers to 56.2% in Generation X and 74.7% in Generation Y/Z and median intake from 9.9 to 14.9 and 57.5 g/day (Kruskal–Wallis *p* < 0.001; Dunn Generation X versus Generation Y/Z *p* < 0.001, FDR q < 0.001). The corresponding mean intakes were 40.6, 34.7 and 78.4 g/day; the mean in Baby Boomer men exceeds that in Generation X men because the small number of consumers in the oldest group (24 of 46) reported unusually large amounts (mean among consumers 77.8 versus 61.8 g/day), and for this reason, the median and the proportion of consumers, both of which increase monotonically, are the more informative summaries here. In women, the proportion of consumers rose monotonically from 20.2% to 35.1% and 51.7% and the mean intake from 11.3 to 25.1 and 41.1 g/day (*p* < 0.001). In absolute terms, the proportion of consumers rose by 22.6 percentage points in men (+43% in relative terms) and by 31.5 points in women (+156%); the rank-biserial correlation between Generation Y/Z and Baby Boomers was r = 0.38 in men and r = 0.33 in women. The gradient was essentially unchanged after an adjustment for survey site (rank-based regression coefficient per generation step: men β = 0.118, *p* < 0.001; women β = 0.078, *p* < 0.001), was reproduced in the rural-district subsample analysed separately (men *p* < 0.001; women *p* < 0.001) and persisted among plausible energy reporters alone (Section 3.9). Median added-sugar intake rose in parallel, from 24.6 to 35.9 g/day in women (*p* = 0.005, q = 0.035) and from 31.3 to 46.0 g/day in men, the latter not reaching significance (*p* = 0.065).

### 3.7. Dietary Patterns

**Figure 5:**
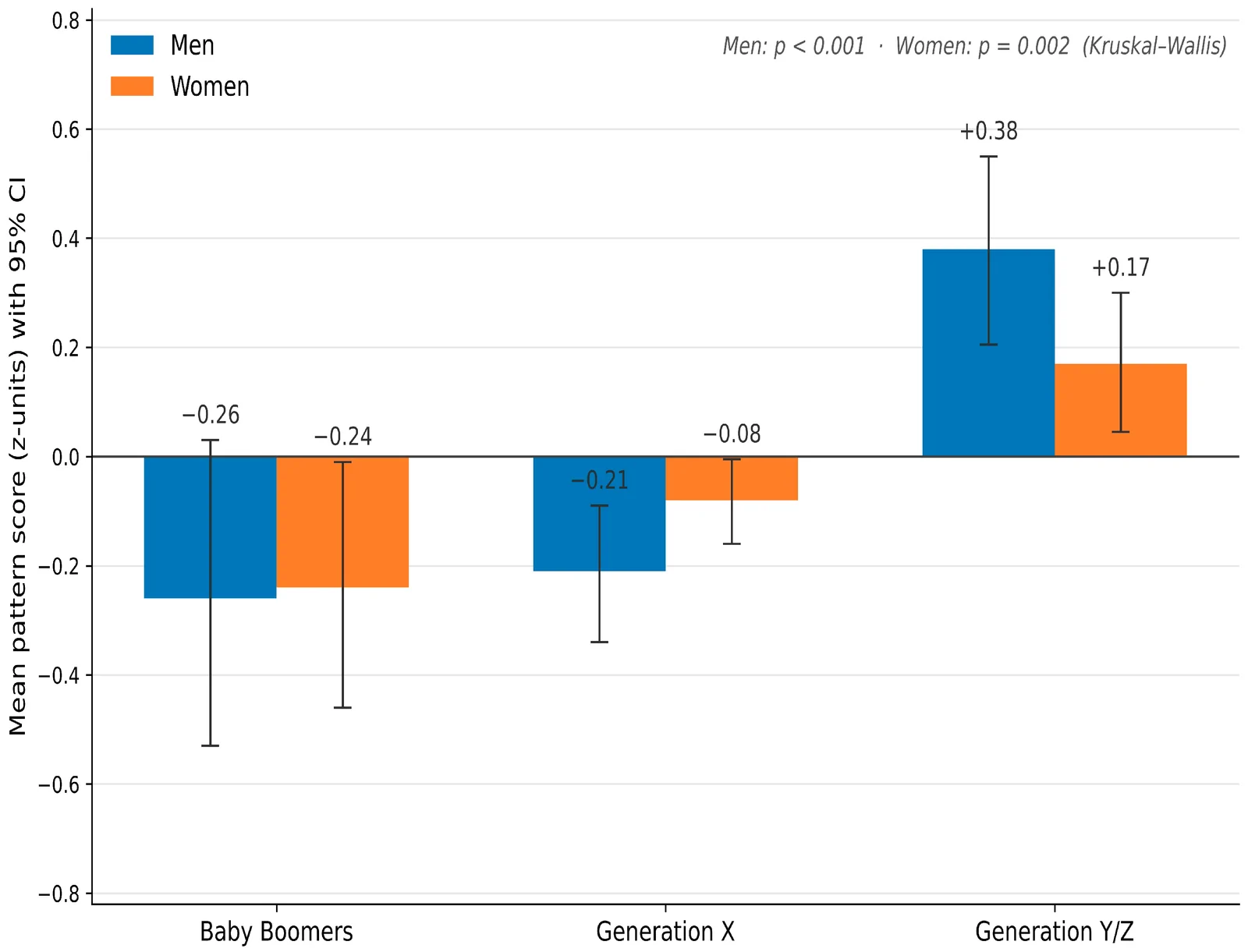
Mean score of the “sweetened-beverage and discretionary foods” dietary pattern (third principal component, 9.9% of variance) by generation and sex. Error bars are 95% confidence intervals obtained via bootstrap resampling (3000 replicates). Higher scores indicate greater intake of sugar-sweetened beverages, table sugar, confectionery and meat and lower intake of bread, cereals, potatoes and vegetables. The generational trend was significant in both sexes (Kruskal–Wallis: men *p* < 0.001; women *p* = 0.002).

Figure 5 shows the generational trend in the dietary-pattern score. Principal component analysis of the 13 energy-adjusted food-group intakes yielded three interpretable components explaining 14.5%, 10.7% and 9.9% of the variance. The first component loaded positively on fruit, dairy products, fish and vegetables and negatively on bread and cereals, table sugar and potatoes, and separated the sexes strongly (*p* < 0.001) but not the generations. The second loaded positively on vegetable oil, vegetables and meat and negatively on dairy products, butter and eggs and likewise distinguished the sexes. The third component loaded positively on sugar-sweetened beverages (0.52), table sugar (0.34), meat (0.31) and confectionery (0.26) and negatively on bread and cereals (−0.46), potatoes (−0.31) and vegetables (−0.25) and was interpreted as a “sweetened-beverage and discretionary foods” pattern opposed to a traditional starch-and-vegetable pattern. The score of this pattern increased monotonically from older to younger generations in both sexes (men −0.26 → −0.21 → +0.38, *p* < 0.001; women −0.24 → −0.08 → +0.17, *p* = 0.002; Figure 5), corresponding to a difference of +0.64 z-units in men and +0.41 z-units in women between the youngest and the oldest generation, and did not differ between the sexes (*p* = 0.448). The generational gradient persisted after an adjustment for survey site (men β = 0.092, *p* < 0.001; women β = 0.042, *p* = 0.005).

### 3.8. Correlations Between Nutrients and Food Groups

**Figure 6:**
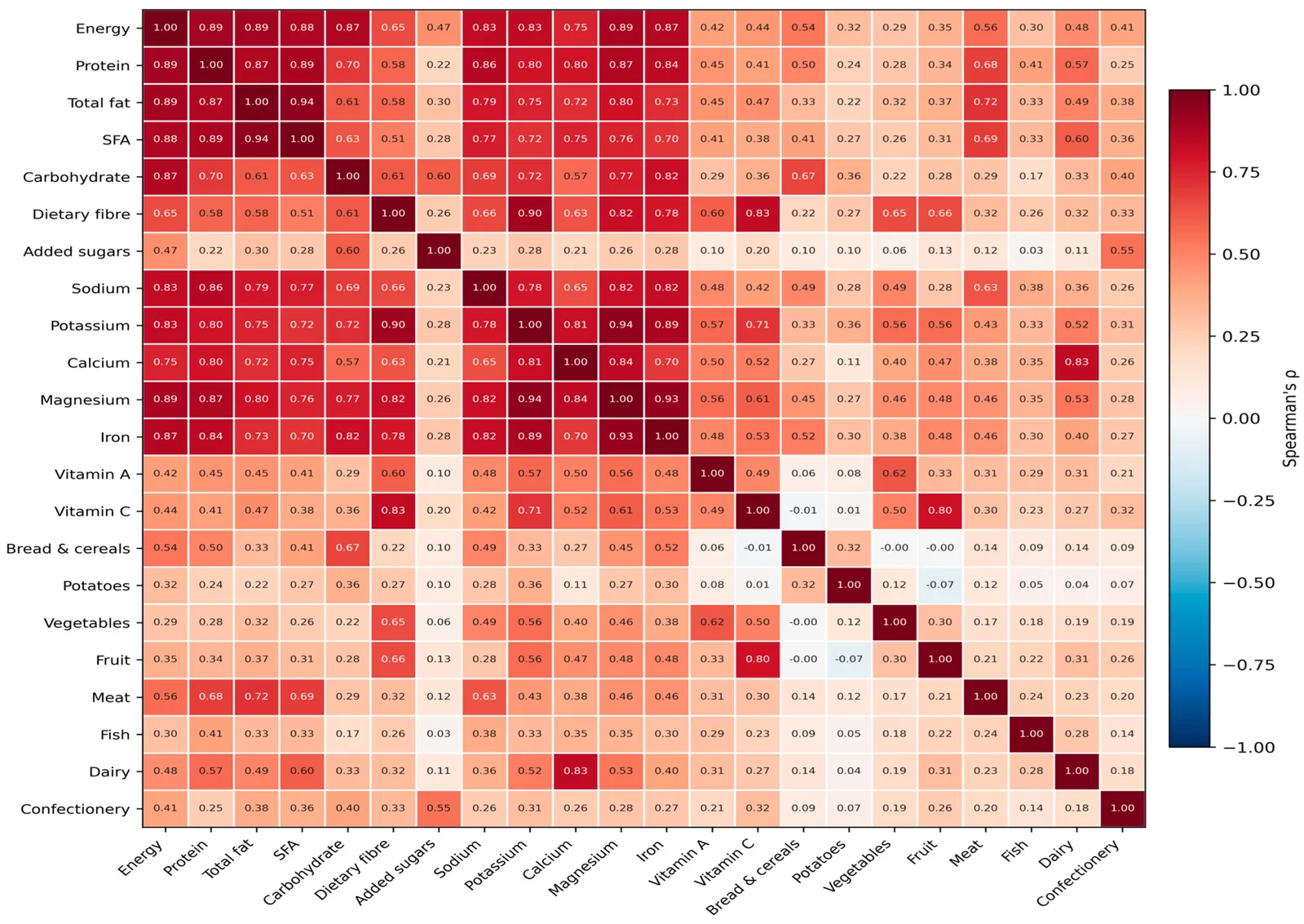
Spearman correlation matrix of nutrient and food-group intakes in the total sample (*n* = 1601). Values are Spearman’s rank correlation coefficients (ρ). Correlations between energy and macronutrients are high because macronutrient intakes are expressed in absolute terms (g/day). Correlations with |ρ| < 0.20 are considered weak and are not interpreted as associations. All shown correlations had *p* < 0.05.

Spearman correlation analysis (Figure 6) showed that dietary fibre intake was determined mainly by vegetables (ρ = 0.65) and fruit (ρ = 0.66) and only weakly by bread and cereals (ρ = 0.22), which suggests that, in this population, this food group consisted mainly of refined products. Calcium intake was closely tied to dairy consumption (ρ = 0.83). Added sugars correlated with confectionery (ρ = 0.55) but were largely independent of the staple food groups (ρ ≤ 0.13 with bread, vegetables, meat and fish), a pattern consistent with discretionary sugars being added to, rather than substituted for, the rest of the diet. Sodium correlated most strongly with meat products (ρ = 0.63) and bread (ρ = 0.49). Vitamin C was almost entirely driven by fruit (ρ = 0.80) and vegetables (ρ = 0.50).

Coefficients with an absolute value below 0.20 are reported for completeness only: in a sample of this size, such values reach statistical significance while accounting for less than 4% of the variance and they are therefore read as evidence of independence between two dietary components rather than as associations. This applies, in particular, to the near-zero correlations between added sugars and the staple food groups, which are interpreted in the Discussion as the absence of substitution.

### 3.9. Sensitivity Analyses

Two sensitivity analyses were performed. First, the sex differences were re-estimated in rank-based models adjusted for survey site and generation. The direction of the difference was preserved for 14 of the 19 variables examined, and the difference remained significant for energy, protein, bread and cereals, potatoes, meat, sodium, iron, sugar-sweetened beverages, total fat as a share of energy, polyunsaturated fatty acids as a share of energy, vegetables and vegetables plus fruit (all *p* ≤ 0.022). Four differences did not survive the adjustment, vitamin C (crude *p* < 0.001, adjusted *p* = 0.517), fruit (*p* < 0.001 to *p* = 0.873), calcium (*p* = 0.005 to *p* = 0.863) and dairy products (*p* = 0.285 to *p* = 0.051), and for confectionery, the direction reversed after the adjustment (crude: higher in women; adjusted: higher in men, *p* = 0.009). These five comparisons are therefore treated as confounded by the place of residence and are not interpreted as sex differences.

Second, the analysis was repeated for the 966 participants (60.3%) whose ratio of reported energy intake to estimated basal metabolic rate lay between 1.35 and 2.4. The generational gradient in sugar-sweetened beverages was unchanged (men 4.9 → 14.9 → 57.5 g/day; women 0 → 0 → 9.9 g/day; both *p* < 0.001), as was the rise in the proportion of consumers (men 50.0 → 73.2%; women 18.8 → 52.7%). The generational differences in starch (men *p* = 0.007; women *p* = 0.001), table sugar in women (*p* = 0.010), added sugars as a share of energy (men *p* = 0.029; women *p* = 0.011) and vitamin C in men (*p* = 0.004) were likewise preserved, as was the gradient in the dietary-pattern score in men (*p* = 0.001). Two findings did not survive the restriction, the generational difference in sodium in men (*p* = 0.153) and the gradient in the dietary-pattern score in women (*p* = 0.130), the latter reflecting the smaller number of women retained in the oldest generation (*n* = 48).

### 3.10. Additional Analyses

The survey wave coincided with survey site: all participants from Ulan-Ude were surveyed in 2022 (*n* = 620), those from the Irkutsk Region in 2023 (*n* = 69) and those from the eight rural districts in 2024 (*n* = 912). The distribution of waves differed by sex and generation: 71.1% of men but only 27.8% of women were surveyed in 2022, and all Baby Boomer women were surveyed in 2024. Within each wave, the proportion of sugar-sweetened beverage consumers and the intake of sugar-sweetened beverages increased from the older to the younger generations: in men surveyed in 2022 (*n* = 288), the proportion of consumers rose from 36.7% to 50.0% and 67.7% (Spearman ρ for intake = 0.27, *p* < 0.001), and the gradient in intake was also present in men surveyed in 2023 (*n* = 69; ρ = 0.50, *p* < 0.001) and in 2024 (*n* = 48; ρ = 0.37, *p* = 0.009); in women surveyed in 2022, all of whom belonged to Generation X or Generation Y/Z (*n* = 332), the proportion rose from 40.4% to 65.9% (ρ = 0.26, *p* < 0.001) and in women surveyed in 2024 (*n* = 864), from 20.2% to 33.1% and 43.6% (ρ = 0.14, *p* < 0.001). Replacing the survey site by the survey wave in the rank-based models did not change the estimates (men β = 0.118, women β = 0.076; both *p* < 0.001). In the logistic regression adjusted for survey site, the odds of being a consumer increased per generation step by a factor of 2.17 (95% CI 1.55–3.05) in men and 1.89 (1.52–2.36) in women. The difference in the proportion of consumers between Generation Y/Z and Baby Boomers was 22.6 percentage points (95% CI 7.3–37.7) in men and 31.5 points (20.9–40.1) in women; for the proportion of men exceeding 10%E from added sugars, it was 23.4 points (10.2–32.1).

Under-reporting differed between generations and was most frequent in Generation Y/Z: EI:BMR was below 1.35 in 26.1%, 24.9% and 41.1% of men (*p* = 0.003) and in 30.9%, 29.8% and 37.1% of women (*p* = 0.050), from the oldest to the youngest generation. Among participants with a plausible EI:BMR, total fat exceeded 30%E in 99.8%, saturated fatty acids exceeded 10%E in 94.9% and dietary fibre was below 20 g/day in 99.2%, proportions practically identical to those in the whole sample.

The fibre content implied by the composition data was 0.28 g per 100 g for bread, pasta and cereals, 0.86 g for potatoes, 0.70 g for vegetables and 1.43 g for fruit. These values are several times lower than the total dietary fibre content of the corresponding foods, which is about 2–7 g/100 g for bread and cereal products and about 1.5–3 g/100 g for potatoes, vegetables and fruit [35], and they are consistent with crude-fibre values. Vegetable oil, with medians of 29.1 g/day in men and 26.7 g/day in women, provided a median of 26.4% of total fat. After the exclusion of fat from vegetable oil, the median contribution of fat to energy was 31.2%E in men and 33.8%E in women, and 70.8% of participants still exceeded 30%E.

When reported energy intake was compared with individually estimated requirements, the proportion of participants with intake below 80% of the requirement ranged across the sex–generation groups from 7.7–18.0% at PAL 1.4 to 15.4–31.7% at PAL 1.6 and 44.6–65.8% at PAL 1.9; at every level, the proportion was highest in Generation Y/Z of both sexes. Among women aged 51 years and over (*n* = 392), 74.7% reported iron intake below the national reference value of 18 mg/day, but only 5.9% below 8 mg/day.

## 4. Discussion

**Figure 7:**
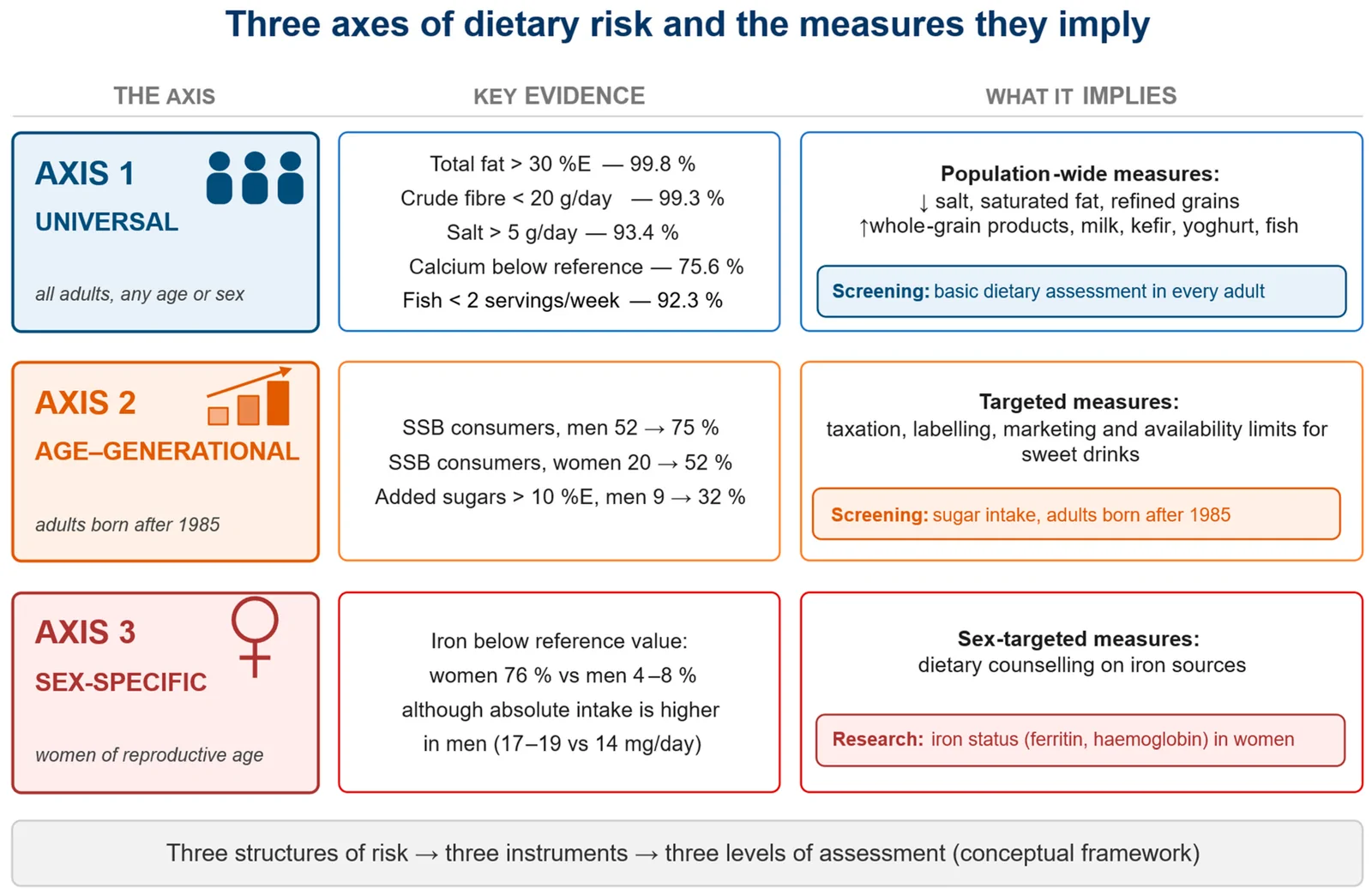
The three axes of dietary risk and the measures that they imply. Each row is read from left to right: the axis and the group it concerns, the key evidence from the present data, and the type of measure and level of screening that follow. The three axes differ in the structure of the risk—a deviation of uniform magnitude, a monotonic gradient across birth-year groups, and a reversal between absolute intake and a sex-specific requirement—and for that reason, they cannot be corrected or detected by the same instrument. The specific measures, the screening level and the evidence supporting each of them are given in Table 7. SSB, sugar-sweetened beverages; %E, percentage of total daily energy.

This analysis of habitual dietary intake in 1601 adults of the Baikal region shows a dietary profile in which the classical nutrition-related risks are shared by the whole population, while one specific risk—discretionary sugars, and above all sweetened beverages—differs markedly between birth-year groups, and a third—low reported iron intake relative to the sex-specific reference value—concerns women. The findings are therefore consistent with a conceptual framework of three axes of dietary risk—universal, age–generational and sex-specific—each of which may call for a different type of preventive measure (Figure 7). The distinction matters, because the three axes are unlikely to be addressed by the same instrument. The framework is derived from a single regional sample and is proposed as a hypothesis-generating structure that requires external validation.

### 4.1. Universal Deviations from Recommendations

Deviations from the macronutrient recommendations of MR 2.3.1.0253-21 were observed in almost every participant and were of similar magnitude in all three generations and both sexes. Total fat exceeded the 30%E limit in 99.8% of participants and saturated fatty acids exceeded 10%E in 95.1%, with a corresponding displacement of carbohydrate to 37.7–42.0%E against a recommended 56–58%E. A high contribution of fat to energy has been reported repeatedly for Siberian and northern populations of Russia, where it has been attributed to culinary practice, to the high fat content of locally produced meat and dairy products and to the limited year-round availability of plant foods [8,9]. Our data add the observation that this profile is similar in birth-year groups born four decades apart, which is more compatible with a durable regional culinary tradition than with recent changes in the food supply, although a single cross-sectional comparison cannot exclude that older and younger adults have converged on a common diet over time. These findings are descriptive and hypothesis-generating: the cross-sectional design precludes causal inference, and what is described here is a deviation of reported intake from reference values, not an observed health outcome.

The apparent shortfall of dietary fibre was the most extreme deviation recorded, although, as discussed below, it is largely attributable to the composition data: a median of 6.1–6.6 g/day, below 20 g/day in 99.3% of participants and below 80% of the reference in 94.7–100%. The correlation structure explains why. Dietary fibre intake tracked vegetables and fruit but only weakly bread and cereals, even though bread products dominated the diet of men by mass (362–406 g/day). In other words, the largest food group in the diet contributed comparatively little to dietary fibre, which reflects, at least in part, the low fibre values assigned to bread and cereal products in the composition data (Section 3.10). A comparable dissociation has been described in Russian population data, where grain products remain a staple while whole-grain consumption is marginal [20]. A shift towards whole-grain bread and cereal products remains consistent with national recommendations [24], but the present data do not allow the size of the fibre deficit to be quantified.

The extreme values for dietary fibre and fat require a consideration of whether they are partly methodological, and the additional analyses (Section 3.10) indicate that for dietary fibre, this is the case. The fibre content implied by the composition data was 0.3 g/100 g for bread, pasta and cereals and 0.7–1.4 g/100 g for potatoes, vegetables and fruit, several times below the total dietary fibre contents of these foods [35] and consistent with crude-fibre values. The reported intake therefore underestimates total dietary fibre intake to a substantial but unknown degree and cannot be compared directly with the reference value of 20–25 g/day, which is defined for total dietary fibre. The near-universal shortfall of dietary fibre shown in Table 2, Table 5 and Table 6 should consequently be regarded largely as an artefact of the composition data rather than as an established deviation; the comparisons of fibre intake between groups, which are not affected by a uniform scaling of composition values, remain valid. A 60-item questionnaire may, in addition, underestimate whole-grain products, legumes, nuts and berries [28]. For fat, the situation is different. Vegetable oil had an unusually narrow distribution (Table 4), which indicates that cooking fat was captured largely through standard portions and it provided about one-quarter of total fat; even after its exclusion, however, the median contribution of fat to energy remained above 30%E in both sexes and 70.8% of participants exceeded this limit, and the proportion with fat above 30%E was unchanged among plausible reporters. The direction of the fat deviation is therefore considered reliable, whereas its magnitude may be overstated. High fat intakes have also been reported in other Siberian and northern populations of Russia [8,9,22]; validation of the questionnaire and of the composition data against repeated 24 h recalls is nevertheless needed before the absolute values are used for quantitative targets.

Sodium intake corresponded to 7.9–10.5 g of salt per day and exceeded the WHO limit in 93.4% of participants, while potassium remained below 3500 mg/day in 68.1% and the Na:K molar ratio exceeded 1 in 98.9%. MR 2.3.1.0253-21 emphasises that the adverse effect of excess sodium is aggravated by the insufficient intake of potassium, magnesium and calcium [24], and all three of these conditions were met simultaneously in this population: magnesium was below the reference in 86.6% and calcium in 75.6%, with a calcium-to-phosphorus ratio of 0.67 against an optimal 1:1. This combination is the dietary configuration most closely linked to hypertension and stroke risk [21,23], and it is relevant that the region has a documented burden of nutrition-related morbidity [22].

### 4.2. The Age–Generational Signal: Sweetened Beverages and Added Sugars

Against this uniform background, one group of foods differed sharply between birth-year groups. As set out in Section 2.2, this gradient is confounded with age by design, so the generational labels used here are descriptive; the comparison with the age-related patterns reported by Guo et al. [14] and Iizuka et al. [15] is made on the understanding that those studies face the same constraint. The proportion of sugar-sweetened beverage consumers was roughly 2.5 times higher in women born after 1985 than in those born before 1963 (51.7% versus 20.2%) and rose by 22 percentage points in men, with mean intake in Generation Y/Z men reaching 78.4 g/day. The proportion of men exceeding the 10%E limit for added sugars increased more than threefold across generations, from 8.7% to 32.1%. The same signal emerged independently from the dietary-pattern analysis: the third principal component, which contrasts sweetened beverages, table sugar and confectionery with bread, potatoes and vegetables, was the only component that ordered the generations, and it did so in both sexes. Principal component analysis does not, however, resolve the age–cohort ambiguity: it identifies combinations of foods that tend to be consumed together, but the difference in the pattern score between groups defined by year of birth is subject to the same confounding by age as any other between-group comparison.

These observations are consistent with generational analyses in other countries. Guo et al. reported, in Chinese adults, that younger generations consumed less cereal and more energy-dense and processed food than their elders [14], and Iizuka et al. found age- and sex-related differences in the diversity of dietary patterns in Japanese adults [15]. The pattern that we describe—a decline of the traditional starch base accompanied by an increase in sweetened beverages—matches the transition described in those settings. It is worth noting that the shift is not simply a substitution: added sugars correlated only weakly with the staple food groups (ρ ≤ 0.13), so in this population, they were added on top of an already fat- and salt-dense diet rather than displacing other foods. This is one plausible mechanism through which a change in one food group can translate into a net increase in metabolic risk. This observation concerns the correlation structure of the diet in the sample as a whole rather than the contrast between groups; it is nevertheless cross-sectional and does not demonstrate the presence or absence of substitution within individuals over time. It also identifies sweetened beverages as a comparatively tractable target, since excise taxation of such beverages is now applied at national level in more than a hundred countries and is regarded by the WHO as an effective instrument for reducing their consumption [36].

The generational gradient in sweetened beverages survived the adjustment for survey site and was reproduced in the rural subsample, which argues against its being an artefact of differential recruitment. It was also present within each survey wave, so it cannot be explained by changes in the food supply between 2022 and 2024. Under-reporting was most frequent in Generation Y/Z (Section 3.10); since under-reporting affects discretionary foods disproportionately [32], the gradient in sweetened beverages is, if anything, underestimated. Two further findings in men point in the same direction as a broader dietary transition: bread, pasta and cereals and starch intake declined in the youngest generation, while vitamin C intake rose, the latter reflecting the higher fruit consumption of younger men. Our earlier integrative analysis of 29,544 adults identified the age periods in which the metabolic complications of obesity accumulate [16]; the present data suggest that one dietary exposure potentially relevant to that accumulation is already concentrated in adults who have not yet reached those ages. That observation is hypothesis-generating with respect to the optimal timing of preventive measures and cannot be tested in a single cross-section. If the difference is largely an age effect, younger adults may reduce their consumption of sweetened beverages as they grow older; if it is largely a cohort effect, the higher consumption may persist as these birth cohorts age. Only repeated surveys of the same population can distinguish between these possibilities.

### 4.3. Sex Differences

Men consumed more energy, protein, bread products, potatoes, sodium, magnesium, phosphorus and iron in absolute terms, while women derived a greater share of energy from fat (46.5–48.0%E versus 41.5–42.5%E) and from polyunsaturated fatty acids and consumed more vegetables, fruit, confectionery and vitamin C. The direction of these differences is consistent with the gender-specific dietary profiles described by Lombardo et al. in relation to cardiometabolic risk [19] and with Russian population data [20,37]. Because the male and female subsamples were recruited in different settings, all of these comparisons were re-estimated with an adjustment for survey site (Section 3.9). The differences in energy, protein, bread and cereals, potatoes, meat, sodium, iron, sweetened beverages, vegetables, vegetables plus fruit and the contribution of total fat and polyunsaturated fatty acids to energy survived adjustment and are interpreted below as sex differences. The differences in vitamin C, fruit, calcium, dairy products and confectionery did not and are treated as confounded by place of residence; they are reported in Table 3 and Table 4 for completeness but are not interpreted.

The most consequential sex difference concerns iron. Absolute iron intake was substantially higher in men than in women in every generation, yet when intake is referred to the sex-specific requirements of MR 2.3.1.0253-21—10 mg/day for men and 18 mg/day for women—the picture reverses: 74.9–77.7% of women reported intake below the reference value against 4.1–7.9% of men, and more than half of women reported intake below 80% of it. Combined with the lower intake of meat relative to men and the very low fish intake in both sexes, this identifies women as the group with the highest probability of inadequate iron intake. These data concern reported intake only. Ferritin, haemoglobin and other markers of iron status were not measured, and the proportion of women with intake below the reference value cannot be equated with the prevalence of iron deficiency [33,34]. Moreover, the reference value of 18 mg/day reflects the requirements of premenopausal women, and its application to postmenopausal women, whose physiological requirement is lower [33], overestimates the proportion with potentially inadequate intake in older women: among women aged 51 years and over, 74.7% reported intake below 18 mg/day but only 5.9% below 8 mg/day, the recommended intake for postmenopausal women in the Dietary Reference Intakes [33]. Whether the low reported intake is accompanied by biochemical iron deficiency in this population remains to be established. Thiamine intake showed a similar sex difference, being below 80% of the reference value in three-quarters of women and in about half of men.

The higher vegetable intake of women translated into a substantially better position based on the WHO 400 g/day criterion for vegetables plus fruit, which was met by roughly two-thirds of women but only one-third of men, and this difference survived the adjustment for survey site. It is one of the few instances in the data where a group achieved an international target, and it suggests that the dietary-fibre shortfall in men could be narrowed by measures directed at vegetable consumption specifically. The apparently higher vitamin C and fruit intake of women, by contrast, did not survive site adjustment and is better read as a rural–urban than as a sex difference.

### 4.4. Public Health Implications

The results are consistent with a differentiated approach to preventive nutrition in the region. Population-wide measures appear warranted for the deviations that affect everyone regardless of generation: reduction of the fat and saturated-fat content of the habitual diet, reformulation and dietary-fibre enrichment of bread products, salt reduction in bread and processed meat and promotion of dairy products to close the calcium gap, and promotion of fish, which was below two servings per week in 92.3% of participants of both sexes. Age- and generation-targeted measures appear to be appropriate for sweetened beverages and added sugars, which are concentrated in adults born after 1985—and whether this concentration reflects a cohort effect, an age effect or both, the target group is the same. Such measures include fiscal policy, front-of-pack labelling, restrictions on marketing and limits on the availability of sweetened beverages in educational and workplace settings; they would be expected to have less effect in older adults, in whom consumption is already low. Two further priorities are suggested by the adequacy analysis: the low reported iron intake of women and vegetable intake in men. The three axes may also imply three levels of dietary assessment: basic assessment of salt, fat, dietary fibre and calcium intake for every adult at the routine preventive examination; targeted assessment of sweetened-beverage and added-sugar intake in adults born after 1985; and, for women, dietary counselling on iron sources combined with research to determine whether the low reported iron intake is accompanied by biochemical iron deficiency. The three axes, the corresponding target groups, the level of screening and the evidence from the present data that supports each measure are summarised in Table 7, and the logic linking them is shown in Figure 7. The proposed measures rest on cross-sectional associations and on the comparison of reported intake with reference values; they require prospective validation and, where possible, biochemical verification, before being adopted as regional policy.

The proposed measures differ in the strength of the evidence on which they rest, and this distinction is indicated in Table 7. The present data directly support the identification of the target groups, since they document the magnitude and distribution of the deviations in this population: this applies to fat, salt, calcium, fish, sweetened beverages and vegetables. For dietary fibre, the present data do not allow the deviation to be quantified (Section 4.1). The choice of instruments—reformulation and salt reduction in bread and processed meat, fiscal measures, front-of-pack labelling and restrictions on the marketing of sweetened beverages—was not evaluated in the present study and rests on existing national and international guidelines and on evidence obtained in other populations [5,24,25,36]. For iron, the present data show only that reported intake in women is low relative to the reference value; a laboratory assessment of iron status is therefore proposed not as a screening recommendation but as a priority for future research, which would establish whether a sex-targeted measure is warranted.

### 4.5. Strengths and Limitations

The strengths of the study are the size of the sample for a regional survey, the simultaneous availability of nutrient and food-group data, the use of current national reference values with age- and sex-specific differentiation and the reporting of results against both national and international criteria. The generational findings were additionally verified through post-hoc pairwise testing with Bonferroni correction, false discovery rate control across the dietary outcomes tested within each sex, an adjustment for survey site with a sensitivity analysis restricted to rural districts and a second sensitivity analysis restricted to participants with a plausible ratio of reported energy intake to the basal metabolic rate. The sex differences were re-estimated with an adjustment for place of residence, and only those that survived adjustment are interpreted.

Limitations. Several limitations should be acknowledged. First, the cross-sectional design means that generation and chronological age are collinear, so the observed differences between birth-year groups may reflect age effects, cohort effects or both and cannot be separated; this applies equally to the differences in dietary-pattern scores derived by principal component analysis. The decline in overweight and obesity across generations illustrates this clearly and is likely to reflect, at least in part, an age effect.

Second, data were collected in three survey waves between 2022 and 2024, a period in which food prices, the availability of particular products and dietary habits may have changed. The survey wave coincided with the survey site (Ulan-Ude in 2022, the Irkutsk Region in 2023 and the rural districts in 2024), so the period and place of residence cannot be separated; an adjustment for the survey site therefore also adjusts for the survey wave, and the gradient in sugar-sweetened beverages was present within each wave (Section 3.10). Because men were surveyed mainly in Ulan-Ude in 2022 and women mainly in rural districts in 2024, the sex comparisons are more exposed to this confounding than the generational comparisons within sex.

Third, dietary intake was self-reported with a food-frequency questionnaire, and misreporting was substantial: 32.5% of participants had EI:BMR below 1.35 and a further 7.1% above 2.4, so that about two in five participants had implausible estimates of energy intake. Under-reporting is often selective, affecting energy-dense and socially less desirable foods more than others [32], and it biases absolute intakes of energy and nutrients downwards. In this sample, under-reporting was most frequent in Generation Y/Z (Section 3.10), which would tend to attenuate rather than create the higher intake of sweetened beverages in this generation; the proportions with intake below reference values are therefore likely to be overestimated and the proportions exceeding upper limits for sugars underestimated. Absolute intakes should consequently be read as approximate, and the conclusions rest primarily on indicators expressed relative to energy and on comparisons between groups. The main between-group findings were preserved in the analysis restricted to plausible reporters (Section 3.9), although that analysis cannot correct for the misreporting that is not reflected in total energy. The dietary fibre values of the composition data correspond to crude rather than total dietary fibre, so absolute fibre intake is substantially underestimated, and standard portions of vegetable oil contributed to the high proportion of energy from fat (Section 3.10 and Section 4.1).

Fourth, the sample is unbalanced by sex (74.7% women) and by generation (57.2% Generation X). Because all generational comparisons were made within sex, the imbalance reduces precision rather than biasing these comparisons, but the estimates for men, and in particular for Baby Boomer men (*n* = 46) and for Baby Boomer women in the analysis restricted to plausible reporters (*n* = 48), are imprecise; the minimum detectable effects for these subgroups are given in Section 2.6. The absence of a significant difference in these subgroups should not be interpreted as evidence of no effect, and the estimates for the oldest generation require confirmation in larger samples.

Fifth, participants were recruited at preventive examinations and community screenings rather than through probability sampling. People attending such examinations may differ from the general population in health awareness, health status and socio-economic position, and the predominance of women indicates differential participation by sex. The sample is therefore not representative of the adult population of the region, and the prevalence estimates cannot be extrapolated to it directly. External validity is further limited by the restriction of the study to the Republic of Buryatia and one adjacent district of the Irkutsk Region, whose climate, food supply, culinary traditions and population composition differ from those of other regions of Russia and of other countries. The three-axis framework is accordingly a conceptual structure derived from one regional sample, and whether the same axes are found elsewhere remains to be tested.

Sixth, physical activity was not measured, and the reference values for energy and macronutrients were assigned using the second physical-activity group (coefficient 1.6) for all participants. If actual physical activity differed from this level, the adequacy of energy, protein, fat and carbohydrate intake expressed in absolute terms would be misclassified, and if it differed systematically by age or sex, the comparisons of absolute adequacy between groups would also be affected. When requirements were estimated individually, the proportion with energy intake below 80% of the requirement varied severalfold with the assumed PAL (from 7.7–18.0% at PAL 1.4 to 44.6–65.8% at PAL 1.9), whereas the ranking of the groups was unchanged (Section 3.10). Indicators expressed as a percentage of energy or per 1000 kcal and the micronutrient reference values do not depend on this assumption. Finally, no biochemical markers of nutritional status were measured, so intake below a reference value indicates potentially inadequate intake and not nutritional deficiency; this applies in particular to iron.

Taken together, these limitations do not alter the main descriptive finding—that deviations from dietary recommendations in this sample can be ordered along universal, age–generational and sex-specific axes—but they define the boundaries within which that finding should be interpreted.

## 5. Conclusions

In 1601 adults from the Baikal region, reported habitual diet deviated from national and international recommendations in almost every participant. The principal findings are the following.

Universal deviations. Total fat exceeded 30%E in 99.8% of participants, saturated fat exceeded 10%E in 95.1%, reported dietary fibre was below 20 g/day in 99.3% (a figure that reflects crude-fibre composition values and overstates the shortfall), salt exceeded 5 g/day in 93.4%, the Na:K molar ratio exceeded 1 in 98.9% and calcium was below the reference value in 75.6%. These deviations were of comparable magnitude in all three birth-year groups and in both sexes; their absolute magnitude should be interpreted in the light of the misreporting and methodological issues discussed in Section 4.5.

Age–generational gradient. The intake of sugar-sweetened beverages and added sugars was the exception. It was markedly higher in participants born in 1985–2005 than in those born in 1943–1963 in both sexes (consumers: 52.2 → 74.7% in men, 20.2 → 51.7% in women; *p* < 0.001, q < 0.001), and it was reflected in a dietary pattern—identified via principal component analysis—in which sweetened beverages, table sugar and confectionery are opposed to the traditional starch-and-vegetable base (Δ = +0.64 and +0.41 z-units). Because age and generation are collinear in this cross-sectional design, this difference may reflect a cohort effect, an age effect or both; the same applies to the difference in the dietary-pattern score, since principal component analysis identifies dietary patterns but cannot separate age from cohort effects.

Sex-specific reversal. Men reported more iron in absolute terms, yet 74.9–77.7% of women reported intake below the sex-specific reference value against 4.1–7.9% of men. In the absence of biochemical markers, this indicates potentially inadequate intake, not iron deficiency. This difference, together with those in energy, protein, bread products, vegetables, sodium and sweetened beverages, persisted after an adjustment for survey site.

Three axes of dietary risk. These findings are consistent with a conceptual three-axis framework of dietary risk—universal, age–generational and sex-specific—in which each axis may call for a different preventive approach: population-wide action on fat, salt, calcium and fish with a basic dietary assessment for all adults; targeted action on sweetened beverages with an assessment of sugar intake in adults born after 1985; and, for women, dietary counselling on iron sources together with research on biochemical iron status. The identification of the target groups rests on the present data, whereas the choice of preventive instruments rests on existing guidelines and on evidence from other populations.

Robustness. The difference between birth-year groups in sweetened-beverage intake was robust to the analytical choices made: it survived Benjamini–Hochberg correction across the dietary outcomes tested in each sex, an adjustment for the survey site or survey wave, analysis within each survey wave, restriction to the rural districts and restriction to the 966 participants (60.3%) with a plausible ratio of reported energy intake to the basal metabolic rate. These analyses address confounding by survey site and misreporting of total energy intake, but not the confounding of age and birth cohort.

Future directions. Prospective studies with biochemical verification of nutritional status—in particular ferritin and haemoglobin in women of reproductive age—are needed to establish whether the dietary differences described here are accompanied by differences in nutritional status. Repeated surveys of the same population would allow age, period and cohort effects to be distinguished, and validation of the questionnaire against repeated 24 h recalls or biomarkers in this population would allow the absolute intakes to be calibrated. The present findings are cross-sectional and hypothesis-generating.

Taken together, these findings identify the structure of dietary risk rather than a single dominant risk factor, and it is that structure—universal, generational and sex-specific—that may guide the choice of preventive instruments. The three-axis framework is proposed as a conceptual structure that requires external validation; it could be tested in other populations with different dietary traditions and age structures.

## Figures and Tables

**Table 1:** Characteristics of the study population by generation and sex (*n* = 1601).

Parameter	Men: Baby Boomers	Men: Gen X	Men: Gen Y/Z	Women: Baby Boomers	Women: Gen X	Women: Gen Y/Z
*n*	46	169	190	94	746	356
Birth years	1947–1963	1964–1984	1985–2005	1949–1963	1964–1984	1985–2005
Age, years	64 [61; 65]	48 [44; 55]	31 [26; 35]	63 [61; 65]	49 [44; 53]	33 [26; 36]
Height, cm	173 [167; 178]	175 [172; 180]	176 [172; 180]	161 [156; 165]	164 [159; 167]	164 [160; 168]
Weight, kg	81.0 [72.0; 93.8]	84.0 [75.0; 92.0]	76.5 [70.0; 85.8]	72.0 [62.2; 81.5]	72.5 [65.0; 82.0]	65.0 [55.8; 75.0]
BMI, kg/m^2^	27.5 [25.7; 29.5]	26.9 [24.7; 29.4]	24.7 [22.1; 27.4]	27.7 [24.5; 31.2]	27.5 [24.4; 30.5]	24.0 [21.0; 27.9]
BMI: Underweight, *n* (%)	0 (0.0)	2 (1.2)	6 (3.2)	0 (0.0)	13 (1.7)	22 (6.2)
BMI: Normal, *n* (%)	10 (21.7)	50 (29.6)	93 (49.2)	26 (27.7)	213 (28.6)	188 (52.8)
BMI: Overweight, *n* (%)	25 (54.3)	83 (49.1)	68 (36.0)	37 (39.4)	303 (40.6)	90 (25.3)
BMI: Obesity, *n* (%)	11 (23.9)	34 (20.1)	22 (11.6)	31 (33.0)	217 (29.1)	56 (15.7)

Data are the median [Q1; Q3] unless stated otherwise. BMI categories follow MR 2.3.1.0253-21 and the WHO classification. BMI, body mass index.

**Table 2:** Energy and macronutrient intake by generation and sex, Me [Q1; Q3].

Parameter	Men: Baby Boomers	Men: Gen X	Men: Gen Y/Z	p 1 (Men)	q 2 (Men)	Women: Baby Boomers	Women: Gen X	Women: Gen Y/Z	p 1 (Women)	q 2 (Women)	p 3 M vs. W: BB	p 3 M vs. W: Gen X	p 3 M vs. W: Gen Y/Z
Energy, kcal/day	2406 [2186; 2824]	2575 [2314; 2932]	2456 [2158; 2812]	0.037	0.079	1956 [1615; 2521]	2158 [1718; 2761]	2089 [1652; 2551]	0.033	0.114	<0.001	<0.001	<0.001
Protein, g/day	75.3 [65.1; 87.9]	80.2 [68.9; 90.9]	74.7 [62.1; 91.5]	0.138	0.218	61.2 [46.0; 77.8]	63.5 [48.3; 81.4]	61.4 [46.9; 81.6]	0.455	0.558	<0.001	<0.001	<0.001
Protein, %E	12.4 [11.7; 13.1]	12.2 [11.4; 12.9]	12.3 [11.3; 13.2]	0.528	0.602	12.1 [10.7; 13.2]	11.8 [10.5; 13.0]	12.0 [10.7; 13.0]	0.166	0.381	0.154	<0.001	0.044
Total fat, g/day	115.5 [98.5; 132.0]	118.9 [104.9; 134.5]	114.5 [99.6; 134.8]	0.201	0.290	103.6 [84.5; 132.9]	108.3 [87.7; 139.7]	105.5 [85.4; 133.2]	0.186	0.381	0.136	<0.001	0.002
Total fat, %E	41.5 [39.1; 45.8]	41.5 [39.5; 44.5]	42.5 [39.8; 46.3]	0.311	0.400	48.0 [43.2; 51.9]	46.5 [42.2; 51.0]	47.0 [42.7; 50.5]	0.096	0.277	<0.001	<0.001	<0.001
SFA, g/day	37.4 [30.5; 44.2]	38.9 [33.0; 45.5]	36.9 [30.7; 45.2]	0.237	0.331	30.3 [23.0; 41.1]	32.8 [25.0; 44.4]	31.8 [23.7; 42.0]	0.087	0.267	0.006	<0.001	<0.001
SFA, %E	13.4 [12.4; 15.4]	13.6 [12.1; 14.7]	13.7 [12.4; 14.8]	0.871	0.890	14.1 [12.1; 15.9]	14.1 [12.4; 15.9]	14.0 [12.5; 15.7]	0.901	0.920	0.637	0.009	0.038
PUFA, g/day	28.4 [25.8; 32.1]	29.4 [27.1; 32.4]	28.0 [25.9; 31.7]	0.027	0.072	28.3 [24.0; 32.6]	28.3 [24.2; 33.5]	27.0 [23.8; 32.0]	0.049	0.161	0.581	0.012	0.027
PUFA, %E	11.0 [9.7; 11.8]	10.3 [9.5; 11.2]	10.5 [9.6; 11.7]	0.279	0.370	12.9 [11.3; 14.7]	12.0 [10.6; 13.8]	12.0 [10.5; 14.0]	0.016	0.074	<0.001	<0.001	<0.001
*n*-6 PUFA, %E	9.98 [8.79; 10.76]	9.29 [8.58; 10.37]	9.50 [8.63; 10.66]	0.200	0.290	11.75 [10.32; 13.60]	11.10 [9.70; 12.69]	11.17 [9.72; 12.81]	0.029	0.111	<0.001	<0.001	<0.001
*n*-3 PUFA, %E	1.11 [0.94; 1.40]	1.06 [0.96; 1.22]	1.07 [0.97; 1.32]	0.514	0.599	1.49 [1.35; 1.75]	1.45 [1.22; 1.78]	1.44 [1.22; 1.74]	0.349	0.475	<0.001	<0.001	<0.001
*n*-6:*n*-3 ratio	9.1 [8.2; 9.6]	9.0 [8.2; 9.4]	8.9 [7.9; 9.5]	0.570	0.635	8.0 [7.4; 8.6]	7.9 [6.4; 8.6]	7.9 [6.9; 8.7]	0.222	0.403	<0.001	<0.001	<0.001
Cholesterol, mg/day	301 [200; 379]	343 [288; 514]	323 [252; 500]	0.037	0.079	217 [147; 323]	254 [157; 361]	248 [155; 354]	0.381	0.491	0.017	<0.001	<0.001
Total carbohydrate, g/day	246.3 [217.1; 290.9]	267.0 [228.9; 304.9]	259.0 [212.9; 293.8]	0.178	0.272	185.5 [141.8; 242.3]	208.4 [157.0; 274.7]	199.2 [146.5; 253.0]	0.017	0.074	<0.001	<0.001	<0.001
Carbohydrate, %E	42.0 [37.7; 45.0]	41.5 [38.2; 44.7]	41.4 [37.4; 45.4]	0.927	0.927	37.7 [32.0; 41.9]	38.9 [33.6; 43.6]	38.3 [33.7; 42.9]	0.202	0.396	<0.001	<0.001	<0.001
Starch, g/day	154.9 [139.3; 189.8]	167.6 [138.5; 189.2]	146.4 [109.8; 171.5]	<0.001	0.002	88.3 [69.9; 125.0]	103.3 [76.0; 131.0]	89.9 [68.1; 123.7]	0.001	0.013	<0.001	<0.001	<0.001
Total sugars, g/day	84.1 [64.1; 117.1]	102.3 [76.2; 126.1]	108.9 [79.9; 135.7]	0.016	0.057	88.6 [60.8; 135.3]	100.6 [67.4; 145.2]	101.3 [65.7; 139.8]	0.187	0.381	0.903	0.956	0.114
Added sugars, g/day	31.3 [15.3; 57.2]	39.8 [23.3; 59.7]	46.0 [23.6; 68.7]	0.065	0.123	24.6 [11.9; 54.0]	36.6 [16.6; 65.1]	35.9 [16.9; 61.0]	0.005	0.035	0.210	0.613	0.014
Added sugars, %E	5.5 [2.9; 8.8]	6.6 [3.6; 9.4]	7.4 [4.0; 10.8]	0.032	0.078	4.5 [2.9; 10.4]	6.8 [3.5; 10.8]	6.6 [3.9; 10.7]	0.011	0.066	0.795	0.118	0.624
Dietary fibre, g/day	6.3 [5.2; 7.7]	6.6 [5.6; 7.6]	6.1 [4.9; 7.3]	0.020	0.057	6.4 [4.6; 9.0]	6.5 [4.6; 9.2]	6.2 [4.5; 9.2]	0.450	0.558	0.768	0.700	0.363
Dietary fibre, g/1000 kcal	2.6 [2.3; 2.9]	2.5 [2.3; 2.9]	2.5 [2.2; 2.9]	0.372	0.456	3.1 [2.7; 4.1]	3.0 [2.5; 3.8]	3.0 [2.4; 4.0]	0.159	0.381	<0.001	<0.001	<0.001

1 Kruskal–Wallis test across generations within sex. 2 q, Benjamini–Hochberg FDR-adjusted p across the set of 49 dietary outcomes reported in Table 2, Table 3 and Table 4 within each sex; q values are given for the generational (Kruskal–Wallis) comparison only, since the post-hoc Dunn tests were already Bonferroni-corrected. 3 Mann–Whitney U test between sexes within generation. BB, Baby Boomers (1943–1963); Gen X, Generation X (1964–1984); Gen Y/Z, Generation Y/Z (1985–2005). %E, percentage of total daily energy; SFA, saturated fatty acids; PUFA, polyunsaturated fatty acids. Reference values are given in Section 2.5.

**Table 3:** Micronutrient intake by generation and sex, Me [Q1; Q3].

Parameter	Men: Baby Boomers	Men: Gen X	Men: Gen Y/Z	p 1 (Men)	q 2 (Men)	Women: Baby Boomers	Women: Gen X	Women: Gen Y/Z	p 1 (Women)	q 2 (Women)	p 3 M vs. W: BB	p 3 M vs. W: Gen X	p 3 M vs. W: Gen Y/Z
Sodium, mg/day	3965 [3408; 4655]	4130 [3610; 4620]	3705 [3105; 4360]	<0.001	0.005	3270 [2732; 4135]	3295 [2560; 4290]	3125 [2430; 4222]	0.178	0.381	0.002	<0.001	<0.001
Salt equivalent, g/day	10.1 [8.7; 11.8]	10.5 [9.2; 11.7]	9.4 [7.9; 11.1]	<0.001	0.005	8.3 [6.9; 10.5]	8.4 [6.5; 10.9]	7.9 [6.2; 10.7]	0.178	0.381	0.002	<0.001	<0.001
Potassium, mg/day	2930 [2650; 3551]	3270 [2811; 3785]	3061 [2583; 3618]	0.019	0.057	2812 [2166; 3541]	2890 [2179; 3806]	2746 [2134; 3763]	0.368	0.488	0.261	<0.001	0.018
Na:K molar ratio	2.31 [2.03; 2.52]	2.09 [1.88; 2.36]	2.06 [1.87; 2.25]	<0.001	0.007	2.04 [1.68; 2.31]	1.96 [1.68; 2.28]	1.95 [1.68; 2.26]	0.623	0.678	<0.001	<0.001	0.002
Calcium, mg/day	708 [549; 890]	810 [649; 989]	789 [606; 970]	0.031	0.078	700 [487; 948]	722 [518; 1047]	702 [501; 998]	0.473	0.565	0.991	0.008	0.042
Magnesium, mg/day	321 [288; 360]	342 [298; 392]	324 [275; 374]	0.018	0.057	292 [219; 347]	286 [220; 368]	271 [213; 359]	0.147	0.381	0.026	<0.001	<0.001
Phosphorus, mg/day	1265 [1087; 1422]	1384 [1186; 1582]	1298 [1044; 1561]	0.014	0.057	1068 [790; 1339]	1099 [820; 1402]	1032 [789; 1398]	0.299	0.457	0.004	<0.001	<0.001
Ca:P ratio	0.58 [0.52; 0.63]	0.60 [0.53; 0.67]	0.62 [0.56; 0.68]	0.018	0.057	0.70 [0.62; 0.76]	0.69 [0.61; 0.77]	0.69 [0.61; 0.76]	0.857	0.893	<0.001	<0.001	<0.001
Iron, mg/day	18.0 [15.5; 19.9]	18.9 [15.9; 21.3]	16.9 [14.5; 19.5]	0.001	0.009	13.9 [11.4; 17.7]	14.1 [11.3; 18.0]	13.6 [10.4; 17.7]	0.281	0.457	<0.001	<0.001	<0.001
Vitamin A, µg RE/day	1227 [894; 1444]	1088 [840; 1378]	972 [759; 1369]	0.074	0.134	1202 [827; 1663]	1212 [814; 1786]	1137 [739; 1693]	0.218	0.403	0.581	0.004	0.011
Thiamine (B1), mg/day	1.21 [1.06; 1.33]	1.22 [1.07; 1.42]	1.13 [0.96; 1.33]	0.013	0.057	0.89 [0.68; 1.07]	0.92 [0.74; 1.20]	0.93 [0.70; 1.18]	0.243	0.426	<0.001	<0.001	<0.001
Riboflavin (B2), mg/day	1.21 [0.96; 1.50]	1.39 [1.16; 1.60]	1.30 [1.04; 1.58]	0.035	0.079	1.13 [0.81; 1.52]	1.19 [0.88; 1.64]	1.13 [0.85; 1.56]	0.290	0.457	0.237	<0.001	0.001
Niacin, mg NE/day	14.6 [13.0; 16.5]	15.1 [13.3; 17.3]	14.1 [12.4; 17.0]	0.116	0.190	11.8 [10.0; 14.3]	12.2 [9.6; 15.3]	12.1 [9.6; 15.0]	0.554	0.632	<0.001	<0.001	<0.001
Vitamin C, mg/day	77.2 [59.5; 115.2]	98.6 [65.2; 131.9]	106.8 [82.1; 144.8]	0.004	0.023	144.3 [89.1; 272.9]	126.4 [83.2; 228.3]	127.7 [86.3; 253.6]	0.315	0.457	<0.001	<0.001	<0.001

1 Kruskal–Wallis test across generations within sex. 2 q, Benjamini–Hochberg FDR-adjusted p across the set of 49 dietary outcomes reported in Table 2, Table 3 and Table 4 within each sex; q values are given for the generational (Kruskal–Wallis) comparison only, since the post-hoc Dunn tests were already Bonferroni-corrected. 3 Mann–Whitney U test between sexes within generation. BB, Baby Boomers (1943–1963); Gen X, Generation X (1964–1984); Gen Y/Z, Generation Y/Z (1985–2005). Salt equivalent = sodium (g) × 2.54. RE, retinol equivalents; NE, niacin equivalents.

**Table 4:** Food-group intake by generation and sex, Me [Q1; Q3].

Parameter	Men: Baby Boomers	Men: Gen X	Men: Gen Y/Z	p 1 (Men)	q 2 (Men)	Women: Baby Boomers	Women: Gen X	Women: Gen Y/Z	p 1 (Women)	q 2 (Women)	p 3 M vs. W: BB	p 3 M vs. W: Gen X	p 3 M vs. W: Gen Y/Z
Bread, pasta and cereals, g/day	397.0 [344.0; 498.8]	405.8 [333.5; 487.8]	362.0 [271.6; 452.1]	<0.001	0.005	231.7 [159.7; 337.6]	267.4 [184.4; 357.8]	241.0 [169.6; 325.0]	0.016	0.074	<0.001	<0.001	<0.001
Potatoes, g/day	114.0 [70.7; 142.5]	114.0 [71.2; 142.5]	106.8 [70.7; 142.5]	0.059	0.121	65.8 [34.5; 124.1]	70.7 [34.5; 117.3]	70.7 [34.5; 106.8]	0.018	0.074	0.010	<0.001	<0.001
Vegetables, g/day	269.7 [215.0; 348.9]	253.7 [218.9; 326.0]	240.6 [162.3; 303.2]	0.014	0.057	381.4 [250.9; 534.4]	368.5 [261.1; 556.5]	349.9 [246.0; 548.0]	0.664	0.708	<0.001	<0.001	<0.001
Fruit, g/day	34.5 [22.2; 106.8]	70.7 [34.5; 160.3]	78.1 [34.5; 123.5]	0.099	0.173	87.9 [41.9; 229.4]	106.8 [34.5; 213.7]	106.8 [41.9; 226.3]	0.484	0.565	<0.001	<0.001	<0.001
Vegetables + fruit, g/day	336.3 [266.7; 391.8]	335.6 [264.7; 428.2]	305.3 [236.4; 412.7]	0.248	0.338	509.3 [327.3; 680.2]	510.6 [346.1; 720.0]	511.8 [341.3; 770.0]	0.975	0.975	<0.001	<0.001	<0.001
Meat and meat products, g/day	107.8 [85.7; 149.7]	122.1 [87.2; 147.4]	117.7 [93.4; 152.4]	0.678	0.738	105.6 [71.5; 147.7]	105.5 [73.2; 152.8]	108.8 [77.8; 151.8]	0.592	0.659	0.306	0.008	0.022
Fish and fish products, g/day	10.7 [7.4; 30.6]	12.7 [7.4; 29.3]	10.7 [5.4; 17.3]	0.009	0.050	10.7 [5.4; 17.0]	10.7 [5.4; 28.5]	9.2 [5.4; 15.0]	0.003	0.023	0.220	0.078	0.490
Milk and dairy products, g/day	129.9 [53.5; 217.6]	166.5 [95.2; 325.7]	176.7 [86.6; 333.8]	0.115	0.190	136.2 [57.0; 342.8]	167.0 [60.0; 344.0]	149.2 [58.2; 328.7]	0.317	0.457	0.490	0.839	0.036
Eggs, units/day	0.59 [0.19; 0.59]	0.59 [0.59; 1.19]	0.59 [0.30; 1.19]	0.064	0.123	0.19 [0.19; 0.59]	0.30 [0.19; 0.59]	0.30 [0.19; 0.59]	0.296	0.457	0.015	<0.001	<0.001
Confectionery, g/day	10.4 [5.7; 24.9]	12.8 [4.3; 25.7]	13.2 [5.7; 26.3]	0.728	0.775	15.4 [7.1; 33.2]	17.4 [8.1; 40.4]	17.1 [8.8; 35.6]	0.330	0.462	0.262	<0.001	0.003
Vegetable oil, g/day	29.1 [26.7; 29.1]	29.1 [26.7; 32.7]	29.1 [26.7; 32.7]	0.778	0.811	26.7 [25.6; 26.7]	26.7 [26.7; 29.1]	26.7 [26.7; 29.1]	<0.001	0.005	<0.001	<0.001	<0.001
Butter, g/day	5.3 [2.6; 10.7]	5.3 [1.7; 10.7]	5.3 [1.7; 10.7]	0.355	0.446	1.7 [1.7; 5.3]	5.3 [1.7; 10.7]	1.7 [1.7; 5.3]	<0.001	0.010	0.034	<0.001	<0.001
Table sugar, g/day	15.5 [5.0; 24.5]	24.5 [5.0; 49.0]	24.5 [5.0; 49.0]	0.398	0.476	1.6 [0.0; 5.0]	5.0 [0.0; 24.5]	5.0 [0.0; 24.5]	0.002	0.016	<0.001	<0.001	<0.001
Sugar-sweetened beverages, g/day	9.9 [0.0; 23.0]	14.9 [0.0; 49.3]	57.5 [2.5; 84.6]	<0.001	<0.001	0.0 [0.0; 0.0]	0.0 [0.0; 23.0]	9.9 [0.0; 28.8]	<0.001	<0.001	<0.001	<0.001	<0.001

1 Kruskal–Wallis test across generations within sex. 2 q, Benjamini–Hochberg FDR-adjusted p across the set of 49 dietary outcomes reported in Table 2, Table 3 and Table 4 within each sex; q values are given for the generational (Kruskal–Wallis) comparison only, since the post-hoc Dunn tests were already Bonferroni-corrected. 3 Mann–Whitney U test between sexes within generation. BB, Baby Boomers (1943–1963); Gen X, Generation X (1964–1984); Gen Y/Z, Generation Y/Z (1985–2005). Values are grams per day except for eggs (units per day).

**Table 5:** Proportion of participants (%) with habitual intake below 80% or above 120% of the age- and sex-specific reference value of MR 2.3.1.0253-21, by generation and sex.

Nutrient	Men: Baby Boomers		Men: Generation X		Men: Generation Y/Z		Women: Baby Boomers		Women: Generation X		Women: Generation Y/Z	
	<80%	>120%	<80%	>120%	<80%	>120%	<80%	>120%	<80%	>120%	<80%	>120%
Energy	15.2	19.6	8.3	19.5	25.3	7.4	20.2	37.2	17.4	39.3	27.8	24.4
Protein	26.1	10.9	17.8	11.8	40.5	8.9	34.0	24.5	30.7	30.6	40.2	22.8
Total fat	0.0	73.9	0.6	80.5	2.6	56.8	0.0	80.9	0.1	84.2	1.1	75.3
Carbohydrate	63.0	2.2	65.1	1.8	81.6	0.0	66.0	8.5	58.2	9.2	70.2	5.3
Dietary fibre	100.0	0.0	99.4	0.0	100.0	0.0	94.7	0.0	95.7	0.0	96.3	0.3
Calcium	76.1	6.5	47.3	8.3	52.1	6.8	64.9	14.9	57.2	15.7	57.6	14.0
Magnesium	58.7	0.0	45.6	2.4	56.8	2.6	72.3	9.6	66.5	6.6	69.1	4.5
Potassium	34.8	2.2	24.3	12.4	38.4	11.1	48.9	18.1	47.1	19.4	50.8	17.1
Phosphorus	0.0	97.8	1.2	96.4	1.1	89.5	4.3	68.1	3.8	72.7	4.8	71.1
Iron	2.2	93.5	1.2	94.1	2.1	86.3	52.1	13.8	52.9	12.3	53.9	11.2
Vitamin A	13.0	67.4	13.6	50.9	20.5	44.2	13.8	64.9	14.6	65.4	16.3	62.4
Thiamine (B1)	45.7	2.2	43.8	0.0	57.9	0.5	83.0	3.2	74.8	0.8	76.4	1.1
Riboflavin (B2)	67.4	2.2	53.3	1.8	63.7	2.6	68.1	9.6	66.4	9.0	68.5	5.9
Niacin	69.6	0.0	60.9	1.8	67.9	1.1	84.0	1.1	77.9	0.9	80.6	0.8
Vitamin C	52.2	21.7	33.1	30.8	23.7	36.8	16.0	57.4	22.4	51.9	21.9	53.7

Columns are grouped by sex and generation; within each group the first column gives the proportion with intake below 80% of the reference value (potentially inadequate intake) and the second the proportion above 120% (potentially excessive intake). Intake below the reference value does not establish nutritional deficiency, since no biochemical markers were measured. Reference values and the source used for each nutrient are given in Section 2.5. Dietary fibre values are based on composition data corresponding to crude rather than total dietary fibre (Section 3.10).

**Table 6:** Proportion of participants (%) not meeting national (MR 2.3.1.0253-21) and WHO dietary criteria, by generation and sex.

Criterion	All	Men: BB	Men: Gen X	Men: Gen Y/Z	Women: BB	Women: Gen X	Women: Gen Y/Z	p 1 (Men)	p 1 (Women)
Total fat > 30%E	99.8	100.0	100.0	100.0	100.0	99.6	99.7	1.000	0.799
SFA > 10%E	95.1	97.8	98.2	97.4	92.6	93.6	96.1	0.860	0.192
Carbohydrate < 50%E	94.7	93.5	96.4	95.3	96.8	93.8	94.9	0.662	0.431
Added sugars > 10%E	28.3	8.7	20.7	32.1	26.6	29.9	29.5	0.001	0.804
Cholesterol > 300 mg/day	44.6	50.0	71.6	63.2	34.0	38.9	36.0	0.018	0.488
Dietary fibre < 20 g/day	99.3	100.0	100.0	100.0	97.9	99.1	99.2	1.000	0.517
Salt > 5 g/day	93.4	100.0	98.8	97.9	92.6	92.4	89.9	0.522	0.362
Sodium > 2000 mg/day	92.6	100.0	98.8	97.4	92.6	91.6	88.5	0.365	0.211
Potassium < 3500 mg/day	68.1	69.6	61.5	70.0	72.3	67.2	71.1	0.212	0.312
Na:K molar ratio > 1	98.9	100.0	100.0	100.0	98.9	98.3	99.2	1.000	0.476
Calcium below reference value	75.6	84.8	77.5	80.0	78.7	73.1	75.6	0.542	0.395
Iron below reference value	58.0	4.3	4.1	7.9	77.7	74.9	76.4	0.288	0.771
Vitamin C < 100 mg/day	41.0	67.4	53.3	41.6	34.0	40.1	35.1	0.003	0.198
Vegetables + fruit < 400 g/day	44.6	78.3	70.4	71.1	38.3	35.0	35.7	0.563	0.814
Fish < 2 servings/week (<40 g/day)	92.3	91.3	88.8	90.5	92.6	92.2	94.9	0.809	0.247
*n*-6:*n*-3 ratio > 10	2.9	6.5	1.8	5.3	1.1	2.7	2.5	0.152	0.640

1 Chi-square test across generations within sex. BB, Baby Boomers; %E, percentage of total daily energy; RNI, recommended nutrient intake. The fish criterion corresponds to two servings per week (<40 g/day). The dietary fibre values are based on composition data corresponding to crude rather than total dietary fibre (Section 3.10), so the proportion below the reference value overstates the shortfall.

**Table 7:** Proposed preventive measures and levels of dietary assessment suggested by the three axes of dietary risk, with the supporting evidence from the present study and the source of evidence for the choice of instrument.

Axis	Measure	Target Group	Screening Level	Supporting Evidence from the Present Data
Population-wide	Reduction of the fat and saturated-fat content of the habitual diet	All adults, both sexes, all generations	Basic screening: salt, fat, dietary fibre and calcium intake assessed at the routine preventive examination	Total fat > 30%E in 99.8% and SFA > 10%E in 95.1% of participants, with no generational difference. Instrument: national guidelines [5,24]
	Dietary-fibre enrichment and a shift to whole-grain flour in bread products	All adults, with priority for men	Basic screening: bread and cereal intake and use of whole-grain products recorded at the preventive examination	Dietary fibre < 20 g/day in 99.3%; bread and cereals were the largest food group by mass in men (362–406 g/day) yet correlated only weakly with dietary fibre intake (ρ = 0.22). Instrument: national and WHO guidelines [5,24,25]. Fibre values are on a crude-fibre basis, so the size of the deficit cannot be established (Section 4.1)
	Salt reduction in bread and processed meat	All adults, with priority for men	Basic screening: use of table salt and of salted and processed foods recorded at the preventive examination	Salt equivalent 9.4–10.5 g/day in men and 7.9–8.4 g/day in women; sodium correlated with meat products (ρ = 0.63) and bread (ρ = 0.49). Instrument: national and WHO guidelines [5,24,25]
	Promotion of milk and dairy products	All adults, and those aged 65 years and over in particular	Basic screening: frequency of dairy consumption recorded at the preventive examination	Calcium below the requirement in 75.6%; Ca:P ratio 0.67 against an optimal 1:1; calcium intake tracked dairy consumption (ρ = 0.83). Instrument: national guidelines [24]
	Promotion of fish consumption	All adults, both sexes	Basic screening: frequency of fish consumption recorded at the preventive examination	Fish intake 9–13 g/day in every group; below the equivalent of two servings per week in 92.3% of participants. Instrument: national guidelines [5,24]
Age- and generation-targeted	Fiscal measures based on sugar-sweetened beverages	Adults born after 1985	Targeted screening: sweetened-beverage intake recorded in adults born after 1985	SSB consumers 74.7% in Generation Y/Z men and 51.7% in Generation Y/Z women; mean intake 78.4 g/day in Generation Y/Z men. Instrument: WHO evidence from other populations [36]
	Front-of-pack labelling and restriction of marketing of sweetened beverages	Adults born after 1985	Targeted screening: added-sugar intake estimated in adults born after 1985	Added sugars above 10%E in 32.1% of Generation Y/Z men against 8.7% of Baby Boomer men. Instrument: WHO and national guidelines [24,25]
	Limits on the availability of sweetened beverages in educational and workplace settings	Students and working-age adults	Targeted screening: sweetened-beverage intake recorded at student and occupational health examinations	The “sweetened-beverage and discretionary foods” pattern score rose by +0.64 z-units in men and +0.41 in women between the oldest and the youngest generation. Instrument: national guidelines [5]
Sex-targeted	Dietary counselling on iron sources; evaluation of iron status in future research	Women of reproductive age	Research priority: ferritin and haemoglobin to be measured in women of reproductive age before any screening recommendation is made	Reported iron intake below the sex-specific reference value in 74.9–77.7% of women against 4.1–7.9% of men; more than half of women below 80% of the reference value; no biochemical markers measured
	Promotion of vegetable consumption	Men of all generations	Basic screening: vegetable and fruit intake recorded in men at the preventive examination	Vegetables plus fruit below 400 g/day in 70.4–78.3% of men against 35.0–38.3% of women; difference robust to adjustment for survey site. Instrument: WHO guidelines [25]

Three levels of screening correspond to the three axes: basic screening of dietary intake for the whole adult population at the routine preventive examination; targeted screening of sweetened-beverage and added-sugar intake in adults born after 1985; and, for women, research on biochemical iron status before any screening recommendation. Target groups are identified from the present data; the choice of instrument rests on the guidelines and evidence cited in the last column. %E, percentage of total daily energy; SFA, saturated fatty acids; SSB, sugar-sweetened beverages; ρ, Spearman rank correlation coefficient. Percentages are taken from Table 5 and Table 6 and correlation coefficients from Figure 6.

## Data Availability

The data presented in this study are available on request from the corresponding author due to patient privacy and ethical restrictions. The data are not publicly available because they contain information that could compromise the privacy of research participants.

## Abbreviations

BB, Baby Boomer; BMI, body mass index; BMR, basal metabolic rate; CI, confidence interval; EI, energy intake; EI:BMR, ratio of reported energy intake to estimated basal metabolic rate; %E, percentage of total daily energy; FDR, false discovery rate; FFQ, food-frequency questionnaire; Me [Q1; Q3], median with first and third quartiles; MR, methodological recommendations; NE, niacin equivalents; PCA, principal component analysis; pp, percentage points; PUFA, polyunsaturated fatty acids; RE, retinol equivalents; RNI, recommended nutrient intake; SFA, saturated fatty acids; SSB, sugar-sweetened beverages; STROBE-nut, Strengthening the Reporting of Observational Studies in Epidemiology—Nutritional Epidemiology; WHO, World Health Organization.
